# Reverberation impairs brainstem temporal representations of voiced vowel sounds: challenging “periodicity-tagged” segregation of competing speech in rooms

**DOI:** 10.3389/fnsys.2014.00248

**Published:** 2015-01-12

**Authors:** Mark Sayles, Arkadiusz Stasiak, Ian M. Winter

**Affiliations:** Centre for the Neural Basis of Hearing, The Physiological Laboratory, Department of Physiology, Development and Neuroscience, University of CambridgeCambridge, UK

**Keywords:** reverberation, pitch, auditory scene analysis, vowel, double vowels, cochlear nucleus

## Abstract

The auditory system typically processes information from concurrently active sound sources (e.g., two voices speaking at once), in the presence of multiple delayed, attenuated and distorted sound-wave reflections (reverberation). Brainstem circuits help segregate these complex acoustic mixtures into “auditory objects.” Psychophysical studies demonstrate a strong interaction between reverberation and fundamental-frequency (F0) modulation, leading to impaired segregation of competing vowels when segregation is on the basis of F0 differences. Neurophysiological studies of complex-sound segregation have concentrated on sounds with steady F0s, in anechoic environments. However, F0 modulation and reverberation are quasi-ubiquitous. We examine the ability of 129 single units in the ventral cochlear nucleus (VCN) of the anesthetized guinea pig to segregate the concurrent synthetic vowel sounds /a/ and /i/, based on temporal discharge patterns under closed-field conditions. We address the effects of added real-room reverberation, F0 modulation, and the interaction of these two factors, on brainstem neural segregation of voiced speech sounds. A firing-rate representation of single-vowels' spectral envelopes is robust to the combination of F0 modulation and reverberation: local firing-rate maxima and minima across the tonotopic array code vowel-formant structure. However, single-vowel F0-related periodicity information in shuffled inter-spike interval distributions is significantly degraded in the combined presence of reverberation and F0 modulation. Hence, segregation of double-vowels' spectral energy into two streams (corresponding to the two vowels), on the basis of temporal discharge patterns, is impaired by reverberation; specifically when F0 is modulated. All unit types (primary-like, chopper, onset) are similarly affected. These results offer neurophysiological insights to perceptual organization of complex acoustic scenes under realistically challenging listening conditions.

## Introduction

Multiple concurrent sound sources are usually present in everyday listening environments. In addition, reverberation is a common feature of most man-made and natural real-world spaces: acoustic pressure waves first arrive at the ears directly from their source(s), followed by *many* delayed, attenuated and distorted indirect waves reflected from nearby surfaces (Sabine, 1922). The auditory system faces the challenge of segregating these jumbled complex pressure waves into perceptually-relevant “auditory objects” (Bregman, 1990). A difference in fundamental frequency (ΔF0) between concurrent complex sounds (e.g., two voices, each with a time-varying F0: the low F0 of an adult male speaker, and the higher F0 of a young child) is a strong cue for this “scene analysis” problem; allowing a target sound to be followed against an interferer under quiet, anechoic conditions (Cherry, 1953; Brokx and Nooteboom, 1982; Scheffers, 1983; Bregman, 1990; Micheyl and Oxenham, 2010). However, this ΔF0-benefit for perceptual segregation is severely diminished in reverberant spaces, especially when F0 is time-varying, as in naturally intonated speech (Culling et al., 1994, 2003; Darwin and Hukin, 2000; Deroche and Culling, 2008, 2011; Lavandier and Culling, 2008).

Pitch-evoking animal vocalizations and the complex sounds produced by musical instruments often consist of a series of harmonics (integer multiples) of a low F0 (e.g., Plack et al., 2005). In the case of voiced speech sounds, F0 typically corresponds to the vocal-fold vibration rate. The F0 of steady-state and time-varying complex sounds is represented in the temporal pattern of action-potential discharge of neurons in the ventral cochlear nucleus (VCN); the first brainstem processing station of the ascending auditory pathway, and an obligatory synapse for all auditory nerve fibers (ANFs) (Rhode, 1995; Sayles and Winter, 2008b). The “residue pitch” of a harmonic complex tone is usually perceived to have the same pitch as a pure tone of frequency F0 (e.g., Moore, 2003; Plack et al., 2005). Temporal representations of F0 based on inter-spike interval distributions are considered a component of the neural processing of pitch-relevant information for many spectrally-diverse complex sounds evoking a common pitch (Cariani and Delgutte, 1996a,b).

Neurophysiological studies of F0-based complex-sound segregation have focused on the ability of ANFs and brainstem neurons in the VCN and inferior colliculus (IC) to represent the two F0s of steady-state concurrent harmonic complexes (Mckinney et al., 2001; Tramo et al., 2001; Sinex and Li, 2007; Larsen et al., 2008; Sinex, 2008; Nakamoto et al., 2010), or concurrent vowel sounds (Palmer, 1988, 1990, 1992; Keilson et al., 1997) under anechoic listening conditions in either their mean firing rates, or their inter-spike interval distributions. Based on VCN single-unit responses to double vowels in the anesthetized cat, Keilson et al. (1997) demonstrated the periodicity of a unit's spike response corresponds to the F0 of the vowel for which the unit's peripheral filter passes more total energy. Therefore, neural synchronization to F0 can be used to apportion firing-rate between the two vowels of a mixture, to reconstruct the spectral profile in an array of band-pass tuned and tonotopically organized neurons. In this way, Keilson et al. argued for a “periodicity-tagged” spectral representation of concurrent vowel sounds in the VCN.

Considering this dual temporal-pattern *and* spike-rate based neural representation, the known detrimental effects of the combination of F0 modulation and reverberation on perceptual segregation of competing voiced speech sounds could result either from disruption of the “periodicity-tagging” process (i.e., impairing the temporal representation of F0 in some way), or by significantly altering the firing-rate profile across frequency. The latter would imply that, regardless of the accuracy with which the F0 could be estimated from temporal discharge patterns, the energy profile reconstructed may result in difficulties of vowel identification when comparing the tonotopic firing-rate profile to some (hypothetical) internally-stored “formant-pattern template” for lexical distinction. Most previous vowel-coding studies have concentrated on the effects of overall sound-pressure level. VCN single units provide relatively stable rate-place representations of vowel spectral envelopes with increasing sound level (Blackburn and Sachs, 1990; May et al., 1998; Recio and Rhode, 2000). This is in contrast to the level-dependent saturation of rate-place representations characteristic of high-spontaneous rate ANFs (Sachs and Young, 1979; Delgutte and Kiang, 1984a; cf., Recio et al., 2002).

The effects of ecologically-relevant reverberation on neural coding have been studied in the context of: (1) representations of the F0 of single harmonic complex sounds, in guinea-pig VCN (Sayles and Winter, 2008b), and in human-brainstem evoked potentials (Bidelman and Krishnan, 2010), and (2) sound-source azimuth location, in the rabbit IC (Devore et al., 2009). Here we examine the neural segregation of concurrent vowel sounds with steady and time-varying F0s, and under anechoic and reverberant listening conditions at a single sound-pressure level, in single-unit responses from the guinea-pig VCN. We present evidence for a neurophysiological correlate of degraded speech segregation in reverberant rooms at the earliest stage of brainstem processing. These data help unravel the neural bases for the detrimental effects of reverberation on ΔF0-based perceptual segregation of concurrent speech.

## Materials and methods

### Ethical approval

The experiments performed in this study were carried out in accordance with the United Kingdom Animals (Scientific Procedures) Act (1986), with approval of the University of Cambridge Animal Welfare Ethical Review Board, and under the terms and conditions of the project license issued by the UK Home Office to Ian M. Winter, and personal licenses issued to Arkadiusz Stasiak, Ian M. Winter, and Mark Sayles. All surgery was performed under anesthesia.

### Animal model

Data described here were collected during experiments performed on pigmented guinea-pigs of either sex, weighing between 300 and 650 g. Animals were anesthetized with urethane (1.0 g/kg, *ip*). Hypnorm (*fentanyl citrate*, 0.315 mg/ml; *fluanisone*, 10 mg/ml; Janssen, High Wycombe, UK) was administered for supplementary analgesia (1 ml/kg, *im*). Anesthesia and analgesia were maintained at a depth sufficient to abolish the pedal withdrawal reflex (front paw). Additional doses of hypnorm (1 ml/kg, *im*) or urethane (0.5 g/kg, *ip*) were administered on indication. Core temperature was monitored with a rectal probe and maintained at 38°C using a feedback-controlled homeothermic blanket (Harvard Apparatus, MA). The trachea was cannulated and, on signs of suppressed spontaneous ventilation, the animal was artificially ventilated with room air via a pump (Bioscience, UK). Surgical preparation and recordings took place in a sound-attenuating chamber (Industrial Acoustics Company). The animal was placed in a stereotaxic frame, which had ear bars coupled to custom-made hollow speculae designed for the guinea-pig ear. A mid-sagittal scalp incision was made and the periosteum and the muscles attached to the temporal and occipital bones were removed. A micromanipulator-mounted silver-coated wire was advanced into the bulla to contact the round window of the cochlea for monitoring tone-evoked compound action potentials (CAPs). The CAP threshold was determined, using an adaptive tracking algorithm, at selected frequencies at the start of the experiment and thereafter upon indication. If thresholds deteriorated by more than 10 dB and were non-recoverable (e.g., by artificially ventilating the animal) the experiment was terminated. A small posterior-fossa craniotomy was performed, exposing the left cerebellum. The dura was resected, and the cerebellum partially aspirated to reveal the underlying cochlear nucleus. A microelectrode was placed, with the tip positioned at the dorsal surface of VCN, under visual guidance with an operating microscope (Zeiss, Germany). After electrode placement, the cerebellotomy defect was filled with 1.5% agar-in-saline to prevent brain desiccation, and to aid recording stability. At the end of the experiment, animals were sacrificed with an overdose of Euthatal (*sodium pentobarbital*, 200 mg/ml, 1 ml *ip*; Merial, Harlow, UK). Death was confirmed by cervical dislocation.

### Neural recordings

Single units were recorded extracellularly with glass-insulated tungsten microelectrodes; impedance of ~0.5 MΩ (Merrill and Ainsworth, 1972). Electrodes were advanced in the sagittal plane, at an angle of 45° to the horizontal plane, through the VCN by a hydraulic microdrive (650 W; David Kopf Instruments, Tujunga, CA). Single units were isolated using broadband noise as a search stimulus. All stimuli were digitally synthesized in real-time with a PC equipped with a DIGI 9636 PCI card that was connected optically to an AD/DA converter (ADI-8 DS, RME audio products, Germany). The AD/DA converter was used for digital-to-analog conversion of the stimuli as well as for analog-to-digital conversion of the amplified (×1000) neural activity. The sample rate was 96 kHz. The AD/DA converter was driven using ASIO (Audio Streaming Input Output) and SDK (Software Developer Kit) from Steinberg.

After digital-to-analog conversion, the stimuli were equalized (phonic graphic equalizer, model EQ 3600; Apple Sound) to compensate for the speaker and coupler frequency response between 50 Hz and 20 kHz, and fed into a power amplifier (Rotel RB971) and a programmable end attenuator (0–75 dB in 5-dB steps, custom built) before being presented over a speaker (Radio Shack 40–1377 tweeter assembled by Mike Ravicz, MIT, Cambridge, MA) mounted in the coupler designed for the ear of a guinea-pig. The stimuli were monitored acoustically using a condenser microphone (Brüel & Kjær 4134, Denmark) attached to a calibrated 1-mm diameter probe tube that was inserted into the speculum close to the eardrum. Neural spikes were discriminated in software (10-μs resolution), stored as spike times on a PC, and analyzed off-line using custom-written MATLAB programs (version 2013b, The MathWorks, Inc., Natick, MA).

### Unit classification

Units were classified on the basis of their pure-tone responses according to the methods of Blackburn and Sachs (1989), and of Young et al. (1988). Upon isolation of a unit, its best frequency (BF) and excitatory threshold were determined using audio-visual criteria. Spontaneous activity was measured over a 10-s period. Single units were classified based on their peri-stimulus time histograms (PSTHs), the first-order inter-spike interval distribution, and the coefficient of variation (CV) of the discharge regularity. The CV was calculated by averaging the ratios of the standard deviation divided by the mean inter-spike interval between 12 and 20-ms after onset (Young et al., 1988). PSTHs were generated from spike times collected in response to 250 presentations of a 50-ms tone at the unit's BF, at 20- and 50-dB above threshold. Tones had 1-ms cos^2^ on and off gates, their starting phase was randomized, and they were repeated with a 250-ms period. PSTHs were classified as primary-like (PL), primary-like with notch (PN), sustained chopper (CS), transient chopper (CT), onset-I (OI), onset-C (OC), or onset-L (OL). For a number of units with very low BFs (<~500 Hz) it was not possible to assign one of the above categories due to very strong phase locking to BF tones, making the detection of chopping peaks near impossible. These units are therefore termed “low frequency” (LF) units.

### Complex stimuli

**Figure 1:**
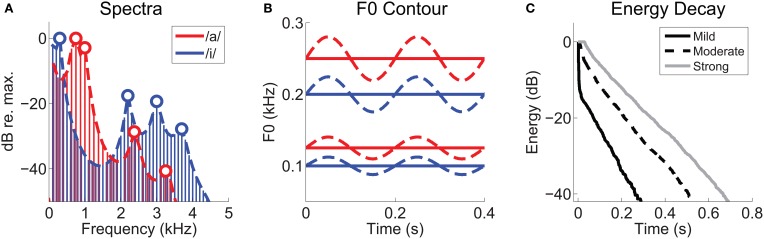
**Synthetic vowel stimuli and reverberation characterization. (A)** Long-term-average magnitude spectra for the vowels /a/ and /i/, synthesized with static F0s of 125 Hz and 100 Hz, respectively. Heavy dashed lines indicate their approximate spectral envelopes. Open symbols indicate the positions of the first four formant frequencies of each vowel. **(B)** Schematic representation of the F0 contours of the synthetic vowel sounds as a function of time. Solid lines represent static F0s, and dashed lines modulated F0s (5-Hz modulation frequency, ±2 semitones). Low-F0 group centered on 100 and 125 Hz. High-F0 group centered on 200 and 250 Hz. **(C)** Energy-decay curves of the three real-room impulse responses, measured at source-receiver distances of 0.32 m (“Mild”), 2.5 m (“Moderate”), and 10 m (“Strong”).

Stimuli were synthetic vowel sounds /a/, and /i/ generated using a Klatt formant synthesizer (Klatt, 1980) implemented in MATLAB (courtesy of Dr Michael Kiefte, Dalhousie University, Canada; http://myweb.dal.ca/mkiefte/). Stimuli were generated at a sampling frequency of 20 kHz, and up-sampled to 96 kHz for presentation. F0 was either static, or sinusoidally modulated at 5 Hz, with a modulation range of ±2 semitones (~12%). This modulation was chosen to allow a direct comparison with the human psychoacoustic findings of Culling and colleagues (Culling et al., 1994). Synthetic /a/ signals were generated with F0s of 125, and 250 Hz. Synthetic /i/ signals were generated with F0s of 100, and 200 Hz. Formant frequencies for /a/ were 0.7, 1.0, 2.4, and 3.3 kHz. For /i/, formant frequencies were 0.3, 2.2, 3.0, and 3.7 kHz (Table 1). All stimuli were 400-ms duration, presented with an 800-ms repetition period in random order for typically 50 presentations, with a new random order on each presentation. Stimuli were gated on and off with 5-ms cos^2^ ramps, and presented at approximately 70-dB SPL. The randomized stimulus array, including all reverberation conditions for the single and double vowel sounds, required 32 minutes of recording time in addition to the time needed for unit characterization using pure-tone stimuli. Therefore, we aimed to collect a large population of well-isolated single-unit responses at one sound level, rather than limit our unit yield in favor of collecting data at multiple sound levels. The aim of the present work is to explore the effects of the reverberation parameter on double-vowel segregation, rather than the effects of overall sound pressure level as has been the case in previous work (e.g., Palmer, 1990). We refer to the vowels synthesized with F0s of 100 and 125 Hz as the “low-F0 vowels,” and those with F0s of 200 and 250 Hz as the “high-F0 vowels.” When referring to specific vowel stimuli, we use the notation /a, i/ (125, 100) to indicate the double vowel consisting of /a/ on an F0 of 125 Hz and /i/ on an F0 of 100 Hz, similar to the notation used by Palmer (1990). Figure 1A shows the long-term average magnitude spectra for the single vowels /a/ and /i/. Vowel F0 as a function of time, is schematized in Figure 1B.

**Table 1:** **Synthetic-vowel formant frequencies and corresponding bandwidths (Hz)**.

	**/a/**	**/i/**	**BW**
F1	700	300	90
F2	1000	2200	110
F3	2400	3000	170
F4	3300	3700	250

Reverberation was added by time-domain convolution with real-room impulse responses recorded in a long corridor using methods previously described in Watkins (2005). These are a subset of the same impulse responses used in our previous work on pitch representations in VCN under reverberant listening conditions (Sayles and Winter, 2008b). Here, we selected three impulse responses, recorded at 0.32, 2.5, and 10 m from a sound source to simulate listening to the vowel sounds at these distances from a speaker in a reverberant room, thereby varying the direct-to-reverberant energy ratios of the final stimuli. We refer to these three levels of reverberation as “mild,” “moderate,” and “strong,” respectively. After convolution the reverberant “tails” introduced at stimulus offset were gated out, and the waveforms were normalized for equal r.m.s. voltage. The energy decay curves, calculated by reverse integration, for each impulse response are plotted in Figure 1C.

### Analyses

#### Correlograms

**Figure 2:**
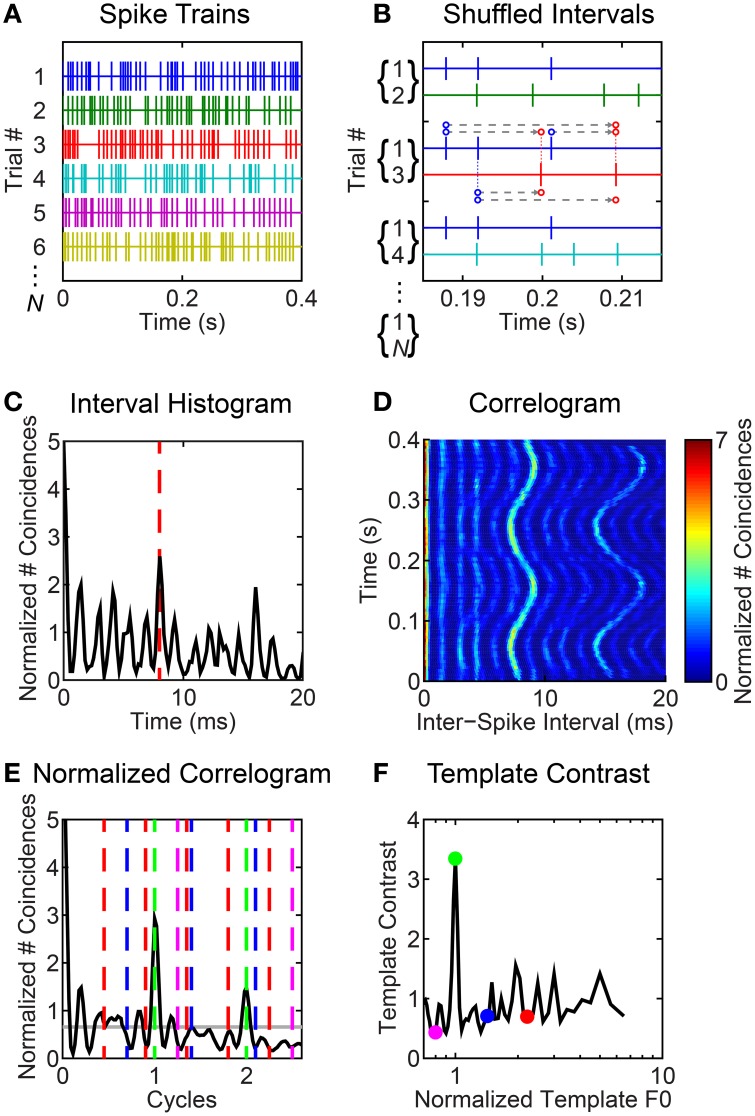
**Illustrated example of inter-spike interval-based analyses of responses to F0-modulated vowels**. Unit was classified as primary-like with notch. *BF* = 1.2 kHz. **(A)** Schematic representation of spike trains in response to multiple repetitions of the vowel /a/ (125) with a modulated F0. Only 6 spike trains are shown, although note we indicate *N* spike trains in total. **(B)** All forward inter-spike intervals are calculated between all non-identical spike-train pairs in a 30-ms sliding window, here centered at 200 ms. In practice, there are *N* (*N* - 1) such spike-train pairs for each windowed segment of the response. All inter-spike intervals are tallied in a histogram. Three ordered spike-train pairs are illustrated. For the middle pair (1,3), we indicate the inter-spike intervals by the dashed gray lines between dots corresponding to each spike. Where necessary due to offset, the dots are connected to their corresponding spike by a thin dashed line. The direction of the intervals is indicated by the arrowheads; i.e., we calculate *forward* inter-spike intervals. **(C)** Inter-spike interval histogram. Red dashed line indicates an inter-spike interval of 8 ms, corresponding to the 125-Hz F0 of the modulated vowel at *t* = 200 ms. **(D)** Correlogram showing the modulated inter-spike interval distribution as a function of time. **(E)** Black line indicates the normalized correlogram. Gray horizontal line indicates the mean of the normalized correlogram. Vertical colored lines indicate the “teeth” of periodic templates used to compute the template-contrast function. **(F)** Template contrast as a function of normalized frequency (1/cycles). Colored symbols correspond to the colored lines in **(E)**. Note that the largest contrast value is at a normalized frequency of 1; i.e., at F0.

Our analyses are based on inter-spike interval distributions derived from the VCN unit spike-trains in response to synthetic vowel stimuli. For clarity, we illustrate the steps in our inter-spike interval analyses for an example unit's response in Figure 2. We compute the *across* spike-train shuffled inter-spike interval distribution in a short rectangular time window (here, 30-ms duration, Figures 2A–C). Figure 2A illustrates spike trains recorded in response to multiple repetitions of the vowel /a/ (125) with a modulated F0. For an analysis window centered at 200 ms, Figure 2B illustrates the calculation of inter-spike intervals between ordered pairs of non-identical spike trains. For this analysis window, the resulting inter-spike interval histogram is plotted in Figure 2C. We slide the analysis window in 5-ms steps through the spike train to estimate the inter-spike interval distribution as a function of time relative to stimulus onset (corrected for response latency and system delay), and plot this as a function of time at the center of the corresponding analysis window (*t*_w,_ Figure 2D). Typically, we recorded responses to 50 repetitions of each stimulus waveform. If fewer than 10 repetitions were collected (occasionally due to recording-time limitations), we discarded the data. In the calculation of the shuffled-interval histograms we included only *across* spike-train intervals. Inter-spike intervals are calculated between each spike in spike-train *A* of a pair (*A,B*) and each *subsequent in time* spike in spike-train *B*. In this sense we consider only *forward* inter-spike intervals between spike-train pairs. It is important to realize that for each ordered spike-train pair (*A,B*), there is the complementary ordered pair (*B,A*). Note in Figure 2B we show ordered spike-train pairs (1,2), (1,3), (1,4)…, (1,*N*). Also included in the analysis but not explicitly in this figure are (2,1), (3,1), (4,1)…, (*N*,1), and *all other possible unique ordered combinations*: hence we have *N*(*N* - 1) spike-train pairs to work with. This is the same shuffling technique as used by Joris et al. (2006), Heinz and colleagues (Kale et al., 2014), and ourselves (Sayles and Winter, 2008b) in recent years. We normalized the interval histograms according to the method of Joris and colleagues, to give the dimensionless units “normalized number of coincidences” (Louage et al., 2004; Joris et al., 2006). The normalization factor for the distribution is *N*(*N* − 1)*r*^2^τw, where *N* is the number of spike trains contributing to each shuffled distribution, *r* is the mean spike rate (spikes s^−1^) in the analysis window, τ is binwidth, and *w* is the analysis-window duration (30 ms). Thus, normalized, we refer to the histograms as *correlograms*. We used methods developed by Delgutte and colleagues to estimate the “pitch contrast” of the correlograms (Larsen et al., 2008; Cedolin and Delgutte, 2010) (Figures 2E,F). Therefore, we calculated the correlograms with 45 bins per “pitch period” (*t* × F0; i.e., units of “cycles”), meaning the absolute binwidth, τ, varies with F0.

#### Pitch templates

We applied a “pitch-sieve” analysis to the time-varying correlograms, to estimate the dominant pitch periods in the inter-spike interval statistics (Cariani and Delgutte, 1996a,b; Cedolin and Delgutte, 2005; Larsen et al., 2008; Bidelman and Heinz, 2011). Periodic templates which select inter-spike intervals at a period and its integer multiples were used to compute *template-contrast* functions from the autocorrelograms (Figures 2E,F). Distinct from previous studies, we applied this temporal pitch-template technique to the responses of single units, rather than to pooled (or pseudo-pooled) population responses. This was possible since the shuffling procedure in the calculation of the correlograms means the number of inter-spike intervals varies as the square of *N*, yielding much smoother histograms than the traditional all-order inter-spike interval histograms used by others (e.g, Cedolin and Delgutte, 2005; Larsen et al., 2008). Template contrast is defined as the ratio of the mean number of coincidences in the histogram bins within the “teeth” of the template “comb” to the mean number of coincidences across all bins of the correlogram. Therefore, a contrast of 1 indicates there is no preferred periodicity in the response. There is no upper bound on the template contrast. Contrast values <1 would indicate a null in the correlogram at the corresponding period and its integer multiples. The template periodicity with the largest contrast was taken as the “pitch match” for the corresponding neural response. Figure 2E shows the mean normalized correlogram calculated from the correlogram in Figure 2D by scaling the abscissa as “cycles” and averaging across windowed time. The colored dashed lines indicate “periodic templates” at which contrast is calculated as indicated by the corresponding colored symbols in Figure 2F. Previous studies have applied an exponential weighting function to the inter-spike interval distribution prior to calculating template contrast, so as to reduce contrast at unrealistically long inter-spike intervals (e.g., Larsen et al., 2008). This was necessary to avoid pitch matches to subharmonic periods, and was supported by the notion of a finite temporal integration window for pitch on the basis of the lower-limit for pitch perception (Pressnitzer et al., 2000). In our implementation, calculating template contrast from inter-spike interval distributions calculated within short temporal windows, we do not require an exponential weighting function, since a similar limitation of contrast at long intervals results from our finite analysis window. The rectangular nature of the analysis window is somewhat arbitrary, but attractive in its simplicity.

#### Statistical analyses

To assess the significance of contrast peaks in the output of the pitch sieve, we implemented a permutation analysis; a form of statistical bootstrap (e.g., Berry et al., 2002; also see Sayles and Winter, 2008a). For each correlogram we generated 1000 randomizations at the input to the pitch sieve. For each bootstrap replication, the correlogram values were randomized without replacement independently for each *t*_w_. The pitch-sieve output was re-computed based on each randomized correlogram. The *p*-value associated with each bin in the observed pitch-sieve output is then the proportion of randomized replications on which the contrast in that bin exceeds the contrast in the observed pitch-sieve output. In practice, for computational efficiency with a large number of bootstrap replications, we only computed *p*-values for bin indices corresponding to a “pitch match.” We consider localized maxima in the pitch-sieve output with associated *p* < 0.01 to be significant.

Analysis of variance (ANOVA) was performed, where appropriate, on data from independent single units using SAS software (version 9.0, SAS institute), with between-unit effects of unit type and BF, and within-unit effects of reverberation, F0 modulation, and F0 range. ANOVAs were constructed as mixed models with a random effect of unit, and a compound-symmetry residual correlation structure to control for repeated measures. Unit BF was included in the models as a continuous variable. Main effects were assessed for significance using the Holm-Bonferroni method, with α = 0.05, to control the family-wise error rate. Where appropriate, pair-wise means comparisons were performed with Holm-Bonferroni correction for multiple comparisons, with α = 0.05.

## Results

We recorded spike times in response to single- and double-vowel sounds, with varying amounts of reverberation, from 129 isolated single units in the guinea-pig VCN. These units were classified as primary-like (PL, 26), primary-like with notch (PN, 10), transient chopper (CT, 38), sustained chopper (CS, 9), onset chopper (OC, 12), onset-I (OI, 4), or onset-L (OL, 8) (see Materials and Methods). A group of units with very low BFs, which cannot be classified into the above categories due to strong phase locking at BF, despite randomization of stimulus starting phase, are termed “low-frequency” units (LF, 19). Three units did not unambiguously fit any of these categories, and are termed “unusual” (UN). For population analyses of responses to vowels, unit classes were grouped together as primary-like (PL/PN), chopper (CT/CS), onset (OC/OI/OL), and low frequency. We targeted the lower-BF regions of the guinea-pig VCN in this study, since the majority of the energy in vowels is at <5 kHz. The median BF (inter-quartile range) for each unit class in this report is: PL/PN, 1.29 kHz (0.734–2.92 kHz); CT/CS, 1.96 kHz (1.28–3.79 kHz); OC/OI/OL, 3.08 kHz (2.21–5.08 kHz); LF, 0.309 kHz (0.196–0.424 kHz).

### Rate-place representations of vowel spectra are robust to F0 modulation and reverberation

We first examine a firing-rate based representation of the two single-vowels' spectral envelopes. This is an important initial step in understanding the effects of reverberation and F0 modulation on the segregation of concurrent vowels, in the context of existing computational models and physiological data. Models of concurrent-vowel segregation are successful at replicating the improvement in segregation with increasing ΔF0 in the range observed in human psychoacoustic studies; i.e., an asymptotic function, with most improvement for ΔF0 between 0 and 2 semitones (Assmann and Summerfield, 1990; Meddis and Hewitt, 1992; De Cheveigné, 1997). In general, these models use some measure of periodicity to assign a proportion of the energy in each frequency band to one or another vowel of the mixture.

**Figure 3:**
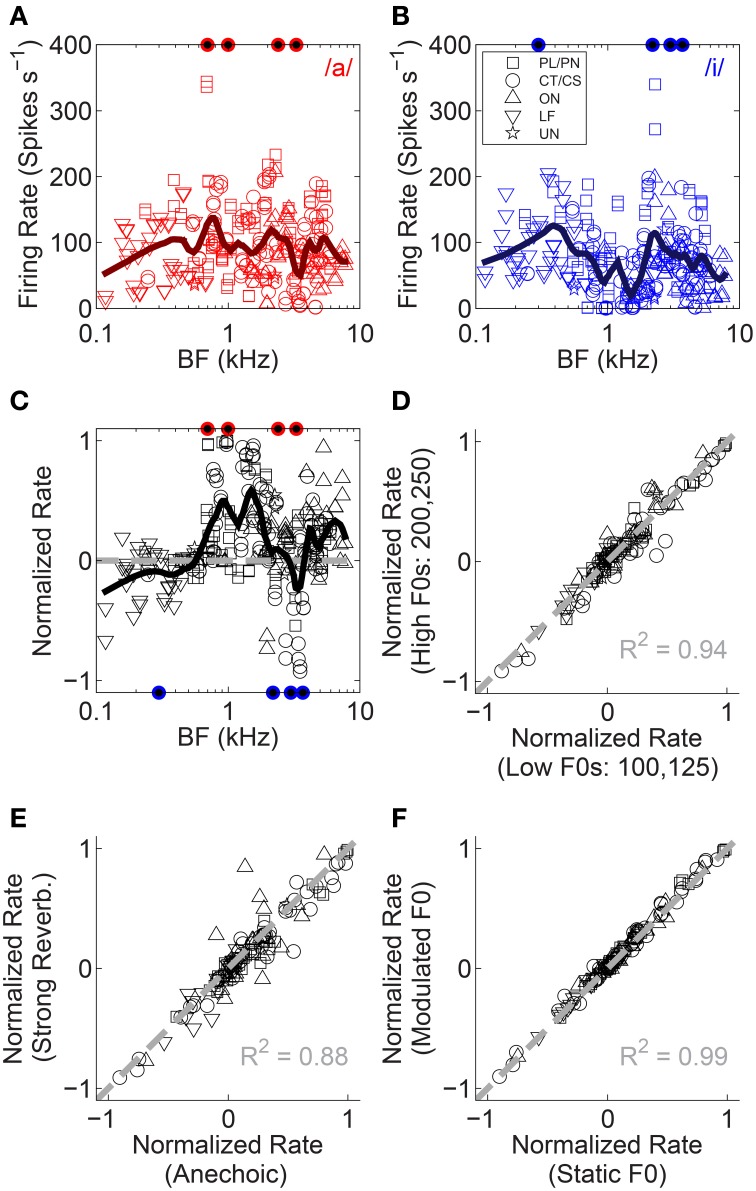
**Normalized firing-rate analyses**. **(A,B)** Raw firing rate as a function of unit BF for both single vowels. Data are plotted for both low-F0 and high-F0 vowels. These data are used to compute the normalized rate (see text). **(C)** Normalized rate as a function of unit BF, for all unit types tested (legend in **B**). Data are based on responses to both the low-F0 and high-F0 vowels. Solid black line represents a *loess* fit to the data to indicate the trend in normalized rate with BF, across all unit types. Gray dashed line indicates a normalized rate of zero (i.e., equal firing rate to /a/ and /i/). Filled symbols along the abscissae indicate the position of the four formant frequencies used in the generation of the synthetic vowels /a/ (upper, red), and /i/ (lower, blue). **(D)** Normalized rate in the high-F0 conditions as a function of normalized rate in the low-F0 conditions. Data plotted are mean data across all levels of reverberation and both modulated and static F0s. **(E)** Normalized rate in the strong reverberation (10-m source-receiver distance) condition as a function of normalized rate in the anechoic condition. Data plotted are mean data across modulated and static F0s and both F0-range conditions. **(F)** Normalized rate in the modulated-F0 conditions as a function of normalized rate in the static-F0 conditions. Data plotted are mean data across all levels of reverberation and both F0-range conditions. **(D–F)**, Gray dashed lines indicate equality. The coefficient of determination (*R*^2^) for equality is displayed in each panel.

The mean firing rate in response to the single vowels /a/ and /i/ is plotted in Figures 3A,B as a function of unit BF. Figure 3C shows the firing rate expressed as a normalized rate which compares a unit's responses to the single vowels /a/ and /i/. This normalized-rate metric is calculated as [*R*_/*a*/_ - *R*_/*i*/_]/[*R*_/*a*/_ + *R*_/*i*/_], where *R* is the mean driven firing rate in response to the corresponding vowel. In contrast to the *within*-vowel normalized-rate metric used by Blackburn and Sachs (1990), our *across*-vowel metric quantifies a unit's differential response to the two single vowels. This is important for further analyses presented herein for double-vowel /a, i/ stimuli. On this normalized-rate scale, normalized rates >0 indicate the unit responds with a higher mean spike rate to /a/ than to /i/, and a normalized rate of 1 would indicate some response to /a/ and an absence of firing in response /i/. Similarly, normalized rates <0 indicate a higher mean spike rate in response to /i/ than to /a/, and a normalized rate of −1 would indicate some response to /i/ and an absence of firing in response to /a/. For clarity, the formant frequencies of the vowels /a/ and /i/ are indicated by filled symbols along the upper and lower abscissae, respectively. The trend across all unit types (black line) indicates the first formant of /i/ is dominant at the lowest BFs. The first two formants of /a/ are dominant for BFs in the region of 1 kHz.

Comparing the responses of single units to vowels synthesized on high F0s with the same vowels synthesized on low F0s, we find the normalized rates for the two are highly correlated (Figure 3D). This is not surprising, since the formant energy peaks remain essentially unchanged with the (small) change in F0. Similarly strong correlation is observed when comparing normalized rates in response to vowels with no reverberation (anechoic), to normalized rates in response to vowels presented in strong reverberation (Figure 3E). The coefficient of determination for the strong reverberation condition is 0.88. For the mild and moderate reverberation conditions this was 0.96 and 0.89, respectively (data not shown). In addition, F0 modulation (at 5 Hz ±2 semitones) is not associated with a change in the normalized firing rates (Figure 3F). These data indicate the VCN across-BF firing-rate profile in response to single vowels /a/ and /i/, is largely independent of F0 range, F0 modulation, and reverberation. Repeated measures ANOVA with the categorical factors F0 modulation (static, modulated), F0 range (low F0s, high F0s), reverberation (anechoic, mild, moderate, strong), and unit type (PL/PN, CT/CS, OC/OI/OL, LF, UN), and with unit BF included as a continuous variable, was performed on the absolute normalized rate data. This indicates a significant main effect of F0 range [*F*_(1, 1898)_ = 16.41, *p* < 0.0001]. Pair-wise means comparison (with Holm-Bonferroni correction for multiple comparisons) indicates significantly higher absolute normalized firing rates in response to the high-F0 conditions compared to the low-F0 conditions [difference of least-squares means (±S.E.) = −0.023 (±0.007), *t*_(1898)_ = −3.42, *p* = 0.006]. There were no other significant simple main effects {reverberation [*F*_(3, 1898)_ = 2.22, *p* = 0.08], F0 modulation [*F*_(1, 1898)_ = 1.12, *p* = 0.29], unit type [*F*_(4, 119)_ = 1.28, *p* = 0.28], BF [*F*_(1, 119)_ = 1.11, *p* = 0.29]}. We also tested two-way interactions between categorical factors, and found no significant interaction between reverberation and F0 modulation [*F*_(3, 1898)_ = 0.26, *p* = 0.85]. We now turn our attention to the effects of reverberation and F0 modulation on the neural representation of stimulus periodicity in inter-spike interval distributions.

### Inter-spike interval representations of single-vowel F0s are degraded by the combination of reverberation and F0 modulation

**Figure 4:**
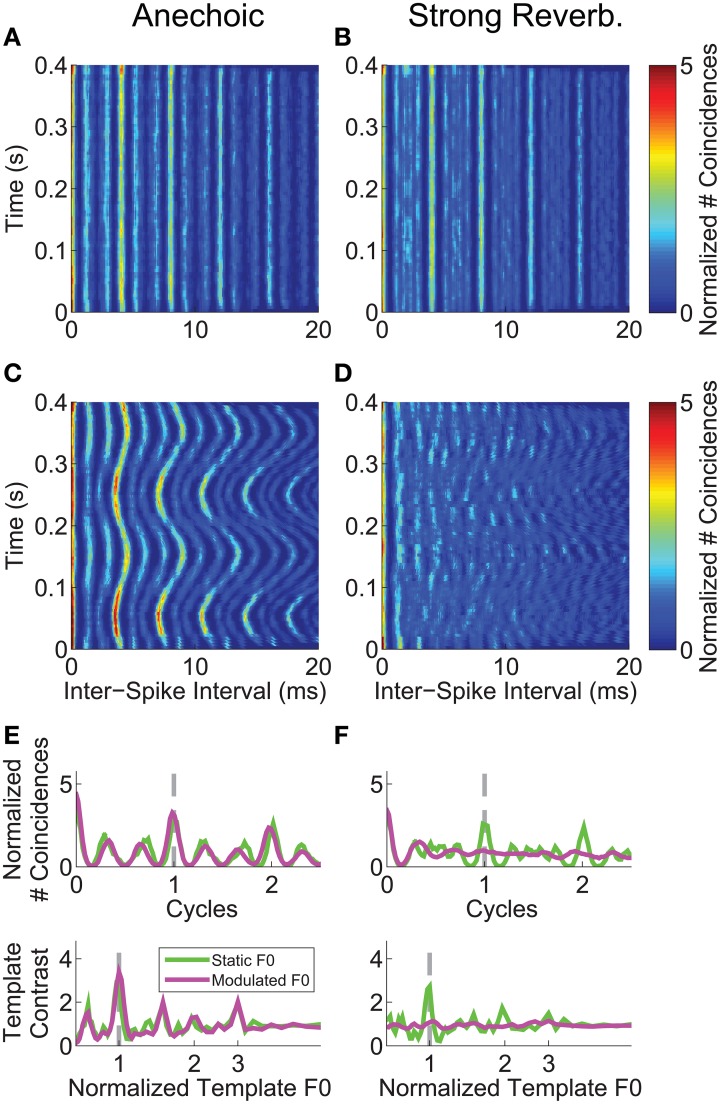
**Inter-spike interval analyses for a single unit's responses to the single vowel /a/ (250-Hz F0)**. Unit was classified as primary-like with notch. *BF* = 1.2 kHz. *Left-hand column*, Responses to vowels with no reverberation (anechoic). *Right-hand column*, Responses to vowels with strong reverberation (10-m source-receiver distance). **(A,B)** Correlograms in response to the single vowel /a/, with a static F0 of 250 Hz. **(C,D)** Correlograms in response to the single vowel /a/, with a modulated F0 centered on 250 Hz, modulated at 5 Hz ±2 semitones. Color-scale bar applies to all correlogram panels in the figure. **(E,F)** Mean F0(*t*)-normalized correlograms (upper plot in each panel), and periodic template-contrast functions (lower plot in each panel). Green solid lines in each plot are calculated from responses to the vowel with a static F0. Magenta solid lines in each plot are calculated from responses to the vowel with a modulated F0. Gray dashed lines indicate 1 cycle (i.e., 1 pitch period) and 1^*^F0 in the upper and lower plots, respectively.

We analyzed the inter-spike interval distribution of the single-unit spike trains in short time windows (30-ms duration) as a function of time relative to stimulus onset, using the same normalization techniques as in our previous work (Sayles and Winter, 2008b), based on methods developed by Joris and colleagues (e.g., Louage et al., 2004; Joris et al., 2006). The correlograms calculated from a single-unit's responses to the vowel /a/ synthesized with a (mean) F0 of 250 Hz, are plotted in Figures 4A–D. This unit was classified as primary-like with notch, with a BF of 1.2 kHz. With a static F0, in both the anechoic and strong reverberation conditions, there is a clear peak (see color scale) in the correlograms at the inter-spike interval corresponding to 1/F0 (4 ms) and its integer multiples. There is also a clear peak in the distribution of inter-spike intervals corresponding to reciprocal of the modulated F0 in the anechoic condition (Figure 4C). In contrast, this pattern is very much diminished in the correlogram based on responses to the vowel with a modulated F0 in the presence of strong reverberation (Figure 4D). The time-dependent inter-spike interval distributions are summarized across time in Figures 4E,F as *summary correlograms* and template-contrast functions (see Materials and Methods). The abscissa for the summary correlograms is scaled as cycles (*t* × F0), meaning that the period corresponding to 1/F0 has a value of 1 on this scale. The major peaks in the normalized correlograms are centered at a value of 1 cycle for the static-F0 and modulated-F0 vowels *without* reverberation, and for the static-F0 vowel *with* reverberation. There is no major peak in the normalized correlogram calculated from the responses of this single unit to the modulated-F0 vowel in strong reverberation. The lower plots in Figures 4E,F show the template-contrast functions at the output of a “pitch sieve” analysis, similar to that implemented in other recent studies of neural segregation of concurrent harmonic complex sounds in the responses of ANFs (Cedolin and Delgutte, 2005; Larsen et al., 2008) (see also, Figure 2). Since the two are closely related, the same pattern of results seen in the summary correlograms is reflected in the template-contrast functions. That is, large peaks corresponding to the stimulus F0 for all conditions except the vowel with a modulated F0 presented in reverberation. In this case, there are no discernable peaks in the template-contrast function. This is an important finding, since template contrast calculated on the basis of temporal structure in inter-spike interval distributions has been shown to correlate with psychophysical pitch strength for a wide range of pitch-evoking sounds (Cariani and Delgutte, 1996a,b). Contrast of 1 indicates no periodicity at the corresponding F0.

**Figure 5:**
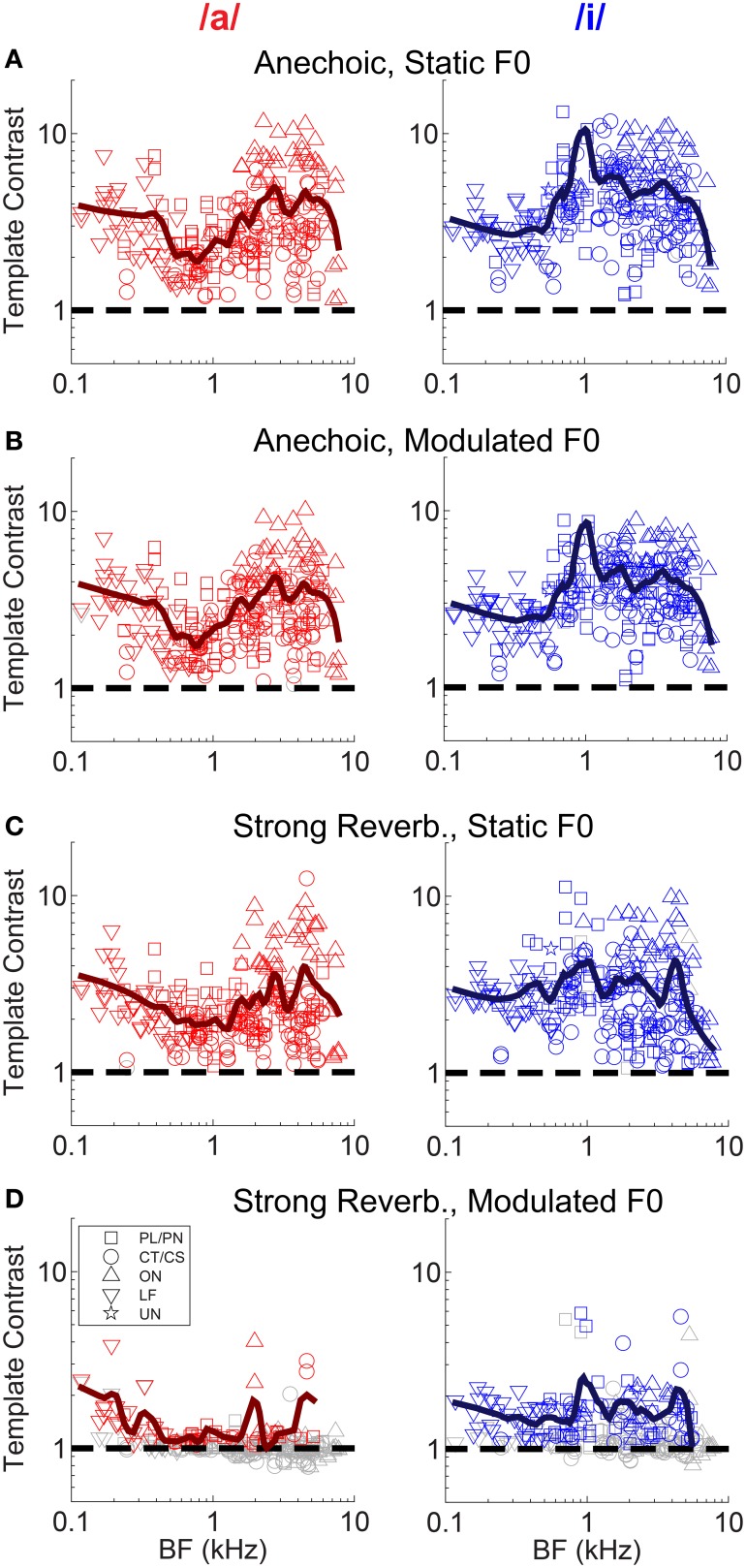
**Template contrast as a function of unit BF, for responses to the single vowels /a/ and /i/**. Data are responses to both low-F0 and high-F0 vowels. **(A)** Static F0, anechoic. **(B)** Modulated F0, anechoic. **(C)** Static F0, strong reverberation. **(D)** Modulated F0, strong reverberation. **(A–D)** Dashed black lines indicate a template contrast of 1, the expected value with a flat correlogram. Colored symbols indicate significant values (permutation test, *p* < 0.01), gray open symbols indicate non-significant values (*p* ≥ 0.01).

Neural mechanisms for complex-sound segregation based on contrast in inter-spike interval distributions would be dependent on both the magnitude of the template contrast, the accuracy with which a peak in contrast estimates the F0 of the sounds to be segregated, and the discriminability of the two estimates. The contrast of the largest peak in the template-contrast function calculated from single-unit responses to the single vowel /a/, is plotted as a function of unit BF in Figure 5. We assessed the statistical significance of peaks in the contrast function using a bootstrap analysis (see Materials and Methods). There is a significant contrast peak in the responses of all units to the vowel /a/ presented with a static F0 without reverberation (Figure 5A). The distribution of template contrast across BF may appear counter-intuitive, since there is a minimum in contrast near to the stimulus spectral-envelope peak corresponding to the first and second formants of /a/ at 0.7 and 1.1 kHz, respectively. However, units with BFs near to these formant frequencies are likely driven to rate saturation by energy in the pass-band of their peripheral filter. This rate saturation likely compresses their representation of the temporal envelope and leads to a relative increase in the denominator for the calculation of template contrast. A strong template contrast is also seen in the majority of units' responses to the modulated-F0 vowel without reverberation, and the static-F0 vowel with reverberation (Figures 5B,C). However, the combination of reverberation and F0 modulation results in a marked reduction in contrast in the units' inter-spike interval distributions (Figure 5D). Only a handful of units maintain a significant contrast peak under these circumstances. The majority of units' contrast peaks are below statistical significance (i.e., *p* ≥ 0.01, permutation test), and cluster around a contrast of 1. These data suggest an *interaction* between F0 modulation and reverberation on the contrast in inter-spike interval distributions, consistent with our previous data on the temporal representation of the F0 of harmonic complex tones with a linear F0 transition (Sayles and Winter, 2008b).

**Figure 6:**
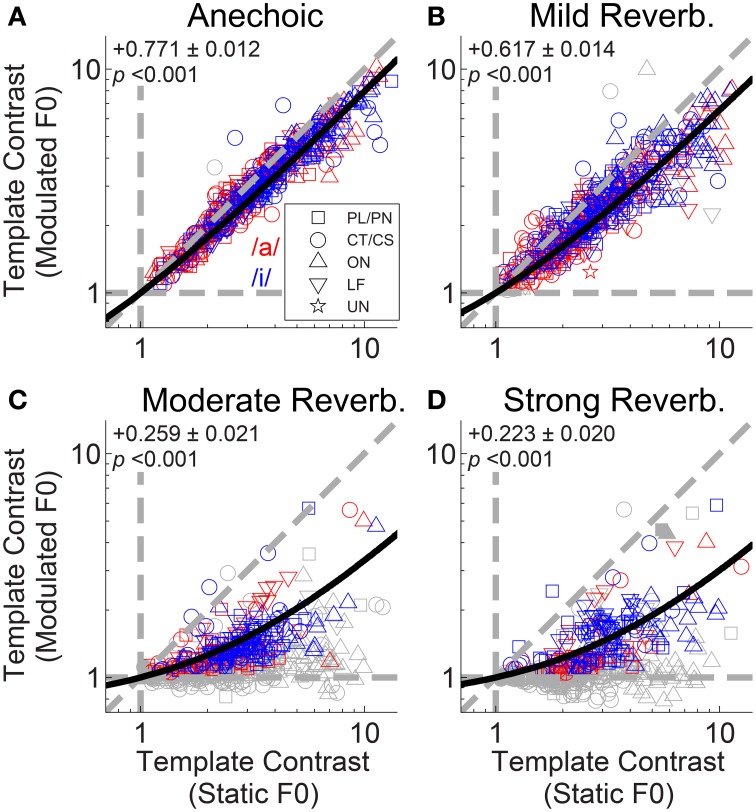
**Template contrast calculated from responses to vowels with modulated F0s, as a function of template contrast calculated from responses to vowels with static F0s: single-vowel responses**. **(A)** Anechoic conditions. **(B)** Mild reverberation. **(C)** Moderate reverberation. **(D)** Strong reverberation. **(A–D)** Colored symbols indicate significant values of contrast for the static and modulated-F0 condition (permutation test, *p* < 0.01). Gray open symbols indicate non-significant values in either static or modulated F0 condition. Gray filled symbols indicate non-significant values in static and modulated F0 conditions. Diagonal gray dashed line indicates equality. Vertical and horizontal gray dashed lines indicate template contrast of 1, for the static and modulated F0s, respectively. Black solid line indicates a linear least squares fit to the significant data (colored symbols). The fit was constrained to pass through (1,1), using an equality constraint in the MATLAB function “*lsqlin*,” from the optimization toolbox. Text in each panel indicates the linear regression slope (β_1_) ± the associated standard error, and the *p*-value for a two-tailed *t*-test with the null hypothesis *H*_0_: β_1_ = 1.

The interaction between F0 modulation and increasing reverberation strength is demonstrated in Figure 6. Template contrast at the largest peak in the contrast function for a modulated-F0 vowel is plotted against template contrast at the largest peak in the contrast function in the responses of that unit to the same vowel with a static F0. In the anechoic condition the data points lie close to the line of equality, meaning there is only a small effect of modulation alone on the template contrast (Figure 6A). However, that said, the linear regression slope is 0.771 (±0.012) and significantly less than 1 (two-tailed *t*-test, *t*_(504)_ = −18.78, *p* < 0.001), indicating there *is* a significant decrease in template contrast due to modulation alone. Some of this decrease may result from the analysis techniques of averaging across multiple short time windows, where essentially the modulated F0 is assumed to be static for the duration of the analysis window (30 ms). Figures 6B–D show the data for mild, moderate and strong reverberation conditions, respectively. As reverberation strength is increased, an increasing number of the units' responses fall below statistical significance (gray symbols), and the linear regression slope of the significant data decreases. Note that the open gray symbols indicate that one of the two contrast peaks failed to reach statistical significance. This is usually the contrast peak (or rather, lack of a peak) in the response to the modulated vowel sound, as evidenced by the clustering of data along the horizontal dashed line at a modulated-F0 contrast of 1 in the moderate and strong reverberation conditions (Figures 6C,D). In such cases, the template contrast in responses to the static-F0 vowel are in the range 1–12, and usually significantly above the noise floor. Repeated measures ANOVA demonstrates significant main effects of reverberation [*F*_(3, 3933)_ = 57.9, *p* < 0.0001] and F0 modulation [*F*_(1, 3933)_ = 151.71, *p* < 0.0001] on template contrast, but no significant main effect of F0 range [*F*_(1, 3933)_ = 0.47, *p* = 0.494]. There is also a significant main effect of unit type [*F*_(4, 118)_ = 4.79, *p* = 0.001]. Pair-wise means comparisons indicate onset units (OC/OI/OL) have significantly higher contrast than PL/PN units [difference of least-squares means (±S.E.) = −1.84 (±0.296), *t*_(118)_ = −6.20, *p* <0.0001] and CT/CS units [−1.69 (±0.307), *t*_(118)_ = −5.50, *p* < 0.0001]. All other pair-wise unit-type comparisons are non-significant; i.e., PL/PN, CT/CS, LF and unusual units are not significantly different in terms of their template contrast. All pair-wise comparisons between different levels of reverberation are significant at *p* < 0.0001, except for the comparison between moderate and strong reverberation [0.117 (± 0.121), *t*_(3933)_ = 0.97, *p* = 1.0]. There are significant two-way interactions between reverberation strength and F0 modulation [*F*_(3, 3945)_ = 24.76, *p* < 0.0001], reverberation strength and unit type [*F*_(12, 3945)_ = 5.43, *p* < 0.0001], and between unit type and F0 modulation [*F*_(4, 3945)_ = 26.8, *p* < 0.0001]. Therefore, we also tested for a three-way interaction term between reverberation strength, unit type, and F0 modulation. This is not significant [*F*_(12, 3933)_ = 1.36, *p* = 0.176].

A group of onset units with BFs between approximately 4 and 6 kHz appear to maintain a relatively high contrast in their inter-spike interval distributions in response to a static-F0 vowel in strong reverberation (Figure 5C). Previous data from our laboratory have shown some onset units are able to maintain a representation of the static F0 of sustained vowel sounds in the presence of background noise, in contrast to VCN CT/CS units (Winter et al., 2003). Therefore, we explored in detail whether VCN onset units are also resistant to the effects of reverberation on their temporal representations of the F0 of the synthetic voiced vowel sounds used here, in terms of template contrast. Considering (Holm-Bonferroni corrected) pair-wise comparisons between the CT/CS and OC/OL/OI groups in response to static-F0 vowels at each level of reverberation, the differences in least-squares means (±S.E.) are: anechoic, −2.23 (±0.327), *t*_(176)_ = −6.83, *p* < 0.0001; mild reverberation, -2.23 (±0.327), *t*_(176)_ = −6.82, *p* < 0.0001; moderate reverberation, −1.47 (±0.327), *t*_(176)_ = −4.51, *p* = 0.001; strong reverberation, −1.41 (±0.327), *t*_(176)_ = −4.3, *p* < 0.0001. Thus, at each level of reverberation the difference between the means of the two unit populations is similar. If onset units were relatively resistant to reverberation, compared to CT/CS units, we would expect the difference between the population means to increase with increasing reverberation strength. We find no evidence for this in our data. However, this does not dispute the finding that in absolute terms our OC/OL/OI units have significantly greater contrast in their inter-spike interval distributions compared to PL/PN and CT/CS units, across reverberation conditions for static-F0 vowels.

**Figure 7:**
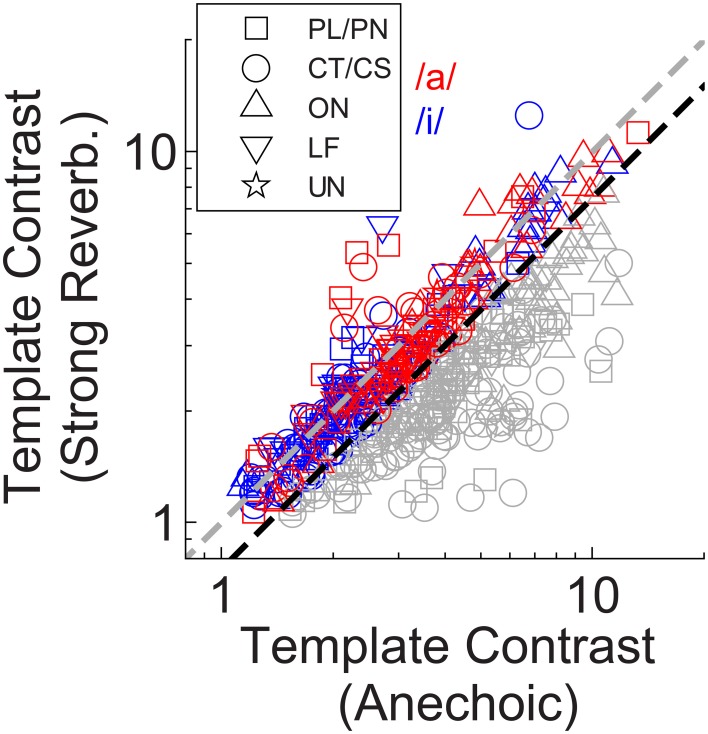
**Template contrast in response to static-F0 vowels: strong reverberation plotted vs. anechoic condition**. Colored symbols indicate those responses for which template contrast in response to the vowel in strong reverberation is ≥ 0.75 times the contrast in response to the same vowel under anechoic conditions. Gray symbols < 0.75 times. Black dashed line has a slope of 0.75. Gray dashed line indicates equality (slope = 1). The choice of 0.75 as a criterion is arbitrary.

A closer examination of the data from Winter et al. (2003) shows 12 of 40 onset units (OC/OL types) maintain a synchronization index >0.5 in response to the steady-state vowel /a/ (with 100 Hz F0) in background noise at 10-dB signal-to-noise ratio. The remaining 28 of 40 onset units in that study behaved similarly to the CT/CS population (synchronization index <0.5 in background noise). Therefore, it is possible a sub-group of onset units exists, which is able to resist the effects of noise (and perhaps reverberation) on temporal coding of the F0 of complex sounds. This sub-group may be “averaged out” in our population statistics presented above, so we now examine the individual unit data in more detail. Figure 7 shows the template contrast in response to static-F0 vowels in strong reverberation plotted against the response to the same static-F0 vowel without reverberation. Note that this analysis considers the relative difference between the reverberation and anechoic conditions, whereas the analyses presented above considered differences in the absolute effect of reverberation on template contrast. Data in Figure 7 are pooled across responses from /a/ (125), /a/ (250), /i/ (100), and /i/ (200). The majority of the data are below the line of equality (gray dashed line), indicating a reduction in template contrast in the strong reverberation condition relative to the anechoic condition. However, a proportion of units' inter-spike interval distributions have contrast values in strong reverberation either equal to, or greater than, those under anechoic conditions. These units are therefore considered relatively resistant to the effects of reverberation on the temporal representation of the F0 of static-F0 vowel sounds. Importantly, there are examples of relatively resistant units of all unit types (PL/PN, CT/CS, OC/OL/OI, LF). We calculated the proportion of units of each type in which the template contrast in response to the vowel in strong reverberation exceeds 0.75 times the contrast in response to the vowel under anechoic conditions (dashed black line). The observed proportions of these “relatively resistant” units (black symbols) does not differ from the expected value (based on the whole population) for CT/CS (*X*^2^ = 3.89, *p* = 0.052), PL/PN (*X*^2^ = 0.11, *p* = 0.736), or OC/OL/OI units (*X*^2^ = 1.74, *p* = 0.187). However, there is evidence for a significantly higher than expected proportion of “reverberation resistant” representations of F0 in the responses of LF units (*X*^2^ = 22.12, *p* < 0.0001).

**Figure 8:**
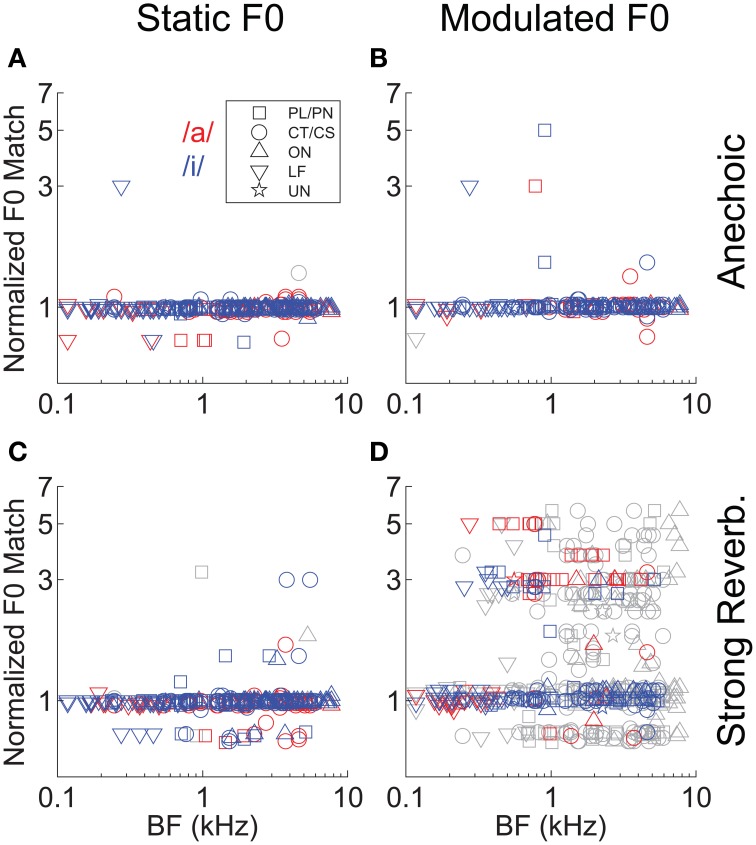
**“Pitch match” as a function of unit BF, in response to single vowels**. **(A)** Anechoic, static F0. **(B)** Anechoic, modulated F0. **(C)** Strong reverberation, static F0. **(D)** Strong reverberation, modulated F0. **(A–D)** Position of the largest localized maximum in the output of the normalized pitch-contrast function. Colored symbols indicate significant localized maxima (permutation test, *p* < 0.01). Gray symbols indicate non-significant localized maxima (permutation test, *p* ≥ 0.01). Note the logarithmic ordinate scale.

In addition to the analyses of template-contrast magnitude, we also considered the implied “F0 match” derived from the normalized F0 template giving the largest peak in the template-contrast function. Figure 8 shows the normalized F0 match derived from the responses of all 129 single units and for each vowel sound. The majority of F0 matches (i.e., the F0 corresponding to the template with the peak contrast at the output of the pitch sieve analysis) are near to a value of 1 on the normalized scale, indicating that in the majority of spike-train responses, the predominant periodicity in the inter-spike interval distributions is at 1/F0. Similar to the analysis of contrast magnitude above, it is the combination of F0 modulation and reverberation which results in an increased variance in the F0 match estimates (Figure 8D).

### “Periodicity tagging” of spectral energy in double vowels is impaired by the combination of reverberation and F0 modulation

**Figure 9:**
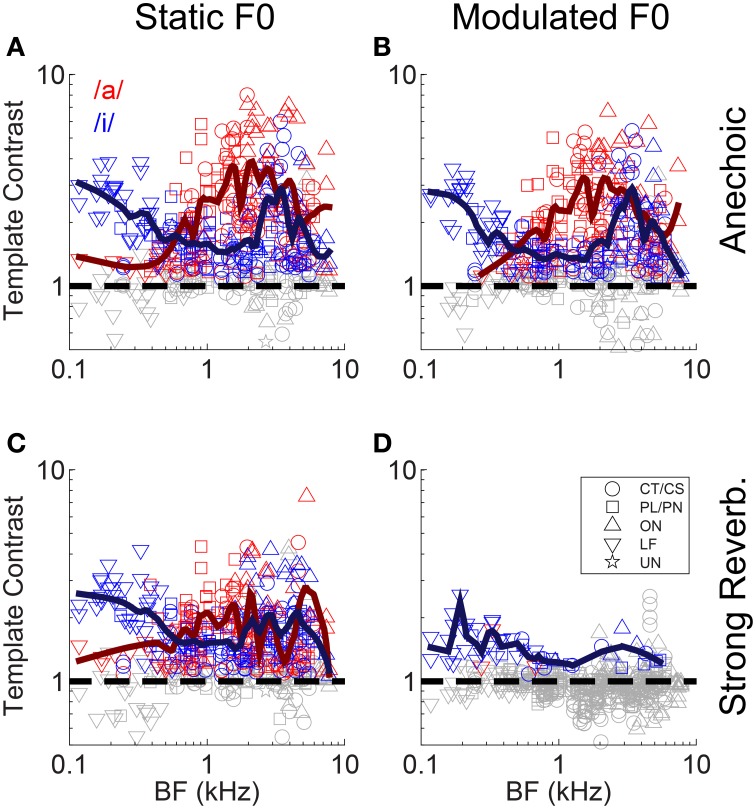
**Template contrast as a function of unit BF, for responses to the double vowel /a, i/**. **(A)** Anechoic, static F0. **(B)** Anechoic, modulated F0. **(C)** Strong reverberation, static F0. **(D)** Strong reverberation, modulated F0. **(A–D)** Red symbols indicate template contrast at the periodicity corresponding to the /a/ component. Blue symbols indicate template contrast at the periodicity corresponding to the /i/ component. Colored symbols indicate significant contrast values (permutation test, *p* < 0.01). Gray symbols indicate non-significant contrast values. Black dashed line is at template contrast of 1.

**Figure 10:**
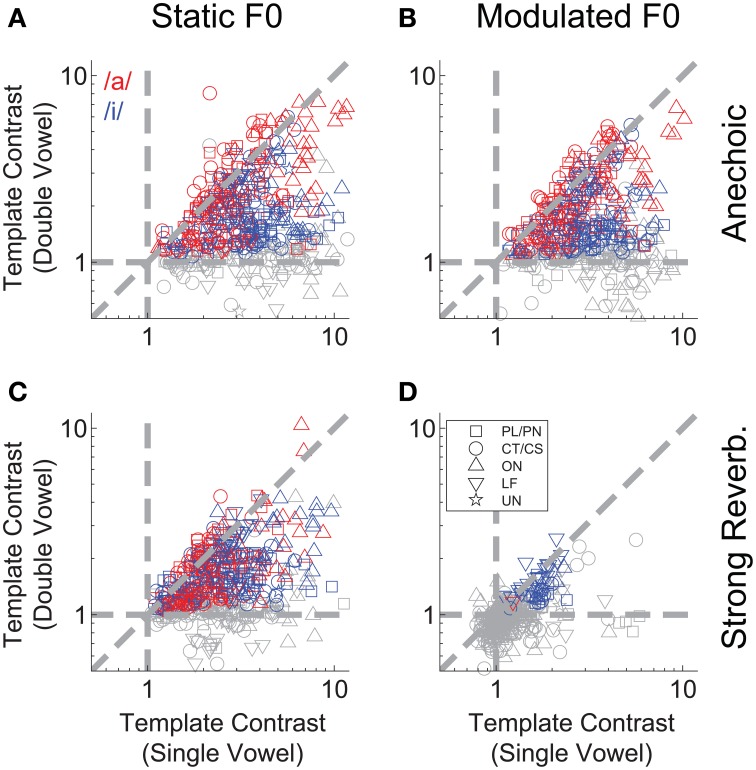
**Double-vowel template contrast as a function of single-vowel template contrast**. **(A)** Anechoic, static F0. **(B)** Anechoic, modulated F0. **(C)** Strong reverberation, static F0. **(D)** Strong reverberation, modulated F0. **(A–D)** Data are plotted for the high-F0 and low-F0 vowels. Red and blue symbols indicate responses to the periodicity of /a/ and /i/, respectively. Gray symbols are non-significant (*p* > 0.01, permutation test). Diagonal dashed gray line indicates equality. Horizontal and vertical dashed lines indicate expected values in the absence of significant template contrast.

We applied the same correlogram and pitch-sieve analyses to the responses of single units to the double vowel /a, i/, with added reverberation, and with either static F0s, or modulated F0s. Template contrast is plotted for both vowels of the double-vowel mixture as a function of unit BF in Figure 9. Responses to the double vowel with static F0s, and without reverberation are shown in Figure 9A. There is a clear effect of unit BF on the differential pattern of response to the two vowels of the mixture. At BFs < ~ 0.5 kHz, units show significant representations of the periodicity corresponding to /i/. That is, in their inter-spike interval distributions, these units are synchronized to the F0 of 100 Hz. The same units tend to have a non-significant template contrast response at the periodicity corresponding to /a/ (gray open symbols in Figure 9A at BFs <~ 0.5 kHz). At 2 kHz < BFs >0.7 kHz, units tend to have a significant representation of the periodicity of both vowels, but a stronger periodicity representation (i.e., higher template contrast) at the periodicity corresponding to /a/ than to /i/. That is, these units tend to be synchronized to both 100 Hz and 125 Hz in their inter-spike interval distributions, but with stronger synchrony to 125 Hz. At BFs >~ 2 kHz, the picture is more mixed, with no clear division between strong representations of the periodicity of /a/ and of /i/. This pattern of results can be understood in terms of the spectral envelopes of the two vowel sounds. The first formant of /i/ is at 0.3 kHz, and the first and second formants of /a/ are at 0.7 kHz and 1 kHz, respectively. Therefore, at very low BFs, units are dominated by the spectral energy peak around the first formant of /i/, and synchronize to the periodicity of that vowel. At BFs between approximately 0.7 and 2 kHz, units are dominated by the first and second formant peaks in /a/, and therefore synchronize to /a/ more than /i/. At higher BFs, the formants of /a/ and /i/ are less well-separated, so the division between them in the units' responses is less clear. This is consistent with the periodicity-tagged representation of double vowel sounds in the cat VCN, as shown by Keilson et al. (1997). In general, template contrast in response to double-vowel stimuli is less than that in response to the corresponding single-vowel stimuli (Figure 10). This is consistent with previous findings in the responses of ANFs to single and double harmonic complex tones (Larsen et al., 2008).

Applying frequency modulation to the F0s of both vowels of the double-vowel mixture (to approximate listening to intonated speech), has very little effect on the template contrast for the two vowel sounds across the tonotopic axis (Figure 9B). The pattern of periodicity across the tonotopic axis is largely preserved, indicating that a mechanism of segregation based on periodicity would be robust to even the relatively high rates of frequency modulation common in natural running speech (e.g., O'shaughnessy and Allen, 1983). With static F0s, the addition of strong reverberation reduces the template contrast, particularly for units with BFs >~1 kHz. This is consistent with the known physical effect of reverberation to reduce amplitude modulation of narrowband-filtered complex sounds, due to the randomization of the phase relationship between component partials (Sabine, 1922; Blauert, 1997; Sayles and Winter, 2008b). However, despite this overall reduction in template contrast, there remains a relatively strong representation of the two vowels' periodicities across BF, with those units with BFs <~0.5 kHz clearly dominated by the periodicity of /i/ (Figure 9C). The combination of F0 modulation and strong reverberation has a dramatic effect on the representation of the vowels' periodicities across BF (Figure 9D). For the majority of units, there is no significant inter-spike interval periodicity information relating to either vowel of the mixture. There is a significant (but weak) representation of the F0 of /i/ in a few LF units with BFs <~0.3 kHz. Therefore, a system which attempts to assign discharge rate to two separate vowels on the basis of common periodicity would struggle under the combination of F0 modulation and the “strong” reverberation used here.

**Figure 11:**
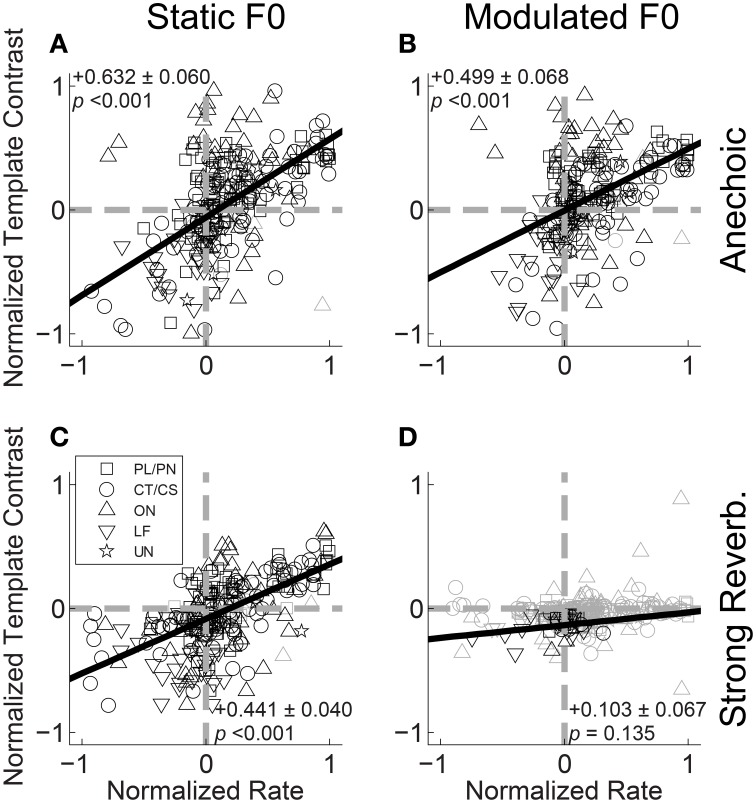
**Normalized template contrast from double-vowel responses plotted against normalized rate from single-vowel responses**. **(A)** Anechoic, static F0. **(B)** Anechoic, modulated F0. **(C)** Strong reverberation, static F0. **(D)** Strong reverberation, modulated F0. **(A–D)** Data are plotted for both low-F0 and high-F0 vowels. Black symbols indicate conditions in which the normalized template contrast was calculated between significant contrast values (*p* < 0.01, permutation test). Gray open symbols indicate conditions in which normalized template contrast was calculated between two non-significant contrast values (i.e., neither the /a/ nor the /i/ contrast was significant in the double-vowel response). Gray dashed lines indicate normalized template contrasts and normalized rates of 0 (i.e., equality between /a/ and /i/). Black solid lines indicate linear least-squares regression fits to the data. Text in each panel indicates the linear regression slope (β_1_) ± the associated standard error, and the *p*-value for a two-tailed *t*-test with the null hypothesis *H*_0_: β_1_ = 0.

If periodicities in the inter-spike interval distribution of a unit's response to a double vowel are to be used by the auditory system to partition discharge rate into two streams for vowel identification, we would predict a positive correlation between the ratio of /a/ periodicity to /i/ periodicity, and the ratio of the same unit's firing rates to the single vowels /a/ and /i/. That is, if a unit fires more spikes in response to /a/ than to /i/ in the single-vowel conditions, we predict a higher template contrast (i.e., more synchrony) at the periodicity of /a/ than at the periodicity of /i/ in the double-vowel responses. This is because the higher firing rate in response to /a/ compared to /i/ indicates (to a first approximation), more energy is passed by the unit's peripheral filter in response to /a/ than to /i/, when the vowels are heard in isolation. Therefore, when they are presented together, a greater proportion of the total energy passed by the filter will originate from the /a/ utterance than from /i/, such that the dominant periodicity in the filter would also correspond to /a/. Figure 11 shows the normalized template contrast [*C*_/*a*/_ − *C*_/*i*/_]/[*C*_/*a*/_ + *C*_/*i*/_] as a function of normalized rate [*R*_/*a*/_ − *R*_/*i*/_]/[*R*_/*a*/_ + *R*_/*i*/_], where *C*_/*a*/_ is the template contrast corresponding to /a/ in the double-vowel response, and *C*_/*i*/_ is the template contrast corresponding to /i/ in the double-vowel response. Both normalized template contrast and normalized rate vary between −1 and 1. A value of −1 on the normalized-rate scale means the unit fires some spikes in response to the single vowel /i/, and no spikes in response to the single vowel /a/. A value of 1 on the normalized-rate scale would indicate the opposite; i.e., some spikes in response to the single vowel /a/, and no spikes in response to the single vowel /i/. In practice, most units respond to both vowels, but with more spikes in response to one of the two, such that most have a normalized rate > −1 and <1. Similarly, a normalized contrast value >0 would indicate more synchrony to /a/ than to /i/ in the double-vowel response. A normalized contrast of <0 indicates more synchrony to /i/ than to /a/. Since the template-contrast function tends to 1 in the absence of a peak in the summary correlogram, in practice the normalized contrast calculated in this way would not reach the extremes of -1 and 1.

Figure 11A plots the data for the responses of all units to /a, i/ (125, 100) and /a, i/ (250, 200) for vowels with static F0s and without reverberation. The solid black line indicates the linear least-squares fit to those data for which the template contrast to at least one of the two vowels of the mixture was significant (permutation test, *p* < 0.01). Although there is a relatively high degree of variance in the data, the slope of the regression line is significantly different from 0 (two-tailed *t*-test, *t*_(236)_ = 10.57, *p* < 0.001), indicating a relationship between normalized contrast and normalized rate in the direction predicted on the basis of the periodicity-tagged spectral representation of concurrent vowels. This positive correlation between normalized contrast and normalized rate is also present in the responses of the single units to the modulated-F0 vowels under anechoic conditions (Figure 11B), and the static-F0 vowels in strong reverberation (Figure 11C) (*p* < 0.001 in both cases). In the combined presence of F0 modulation and strong reverberation, the majority of data are non-significant. From the few units providing a significant representation of vowel-related periodicity in their temporal discharge patterns, the slope of the line relating normalized contrast to normalized rate is not significantly different from 0 (Figure 11D, two-tailed *t*-test, *t*_(31)_ = 1.54, *p* = 0.135). Therefore, for a concurrent-vowel sound with both F0s (co)-modulated at 5 Hz by ±2 semitones, and with the “strong” reverberation studied here (source-receiver distance of 10 m), the inter-spike interval distributions of VCN single units do not contain sufficient information to partition discharge rate into two streams corresponding to the two vowels of the double-vowel mixture /a, i/.

**Figure 12:**
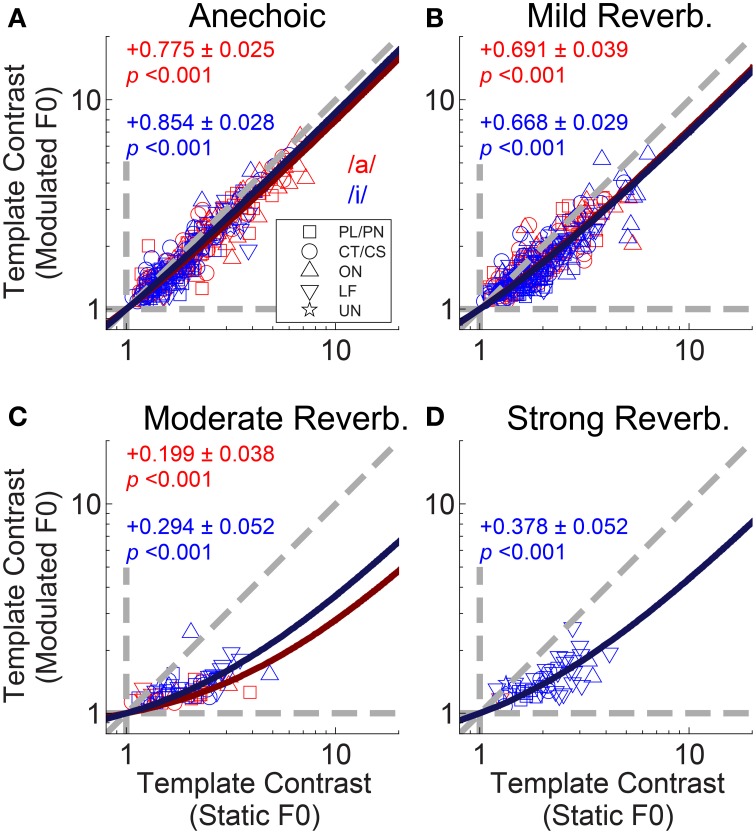
**Template contrast calculated from responses to vowels with modulated F0s, as a function of template contrast calculated from responses to vowels with static F0s: double-vowel responses**. For clarity, only data from those units with significant template contrast in the modulated and static F0 conditions are plotted. **(A)** Anechoic conditions. **(B)** Mild reverberation. **(C)** Moderate reverberation. **(D)** Strong reverberation. **(A–D)** Red symbols indicate responses to the /a/ component. Blue symbols indicate responses to the /i/ component. Diagonal gray dashed line indicates equality. Vertical and horizontal gray dashed lines indicate template contrast of 1, for the static and modulated F0s, respectively. Blue solid line indicates a linear least squares fit to the /i/ responses. Red solid line indicates a linear least squares fit to the /a/ responses. The fits were constrained to pass through (1,1), using an equality constraint in the MATLAB function “*lsqlin*,” from the optimization toolbox. Text in each panel indicates the linear regression slope (β_1_) ± the associated standard error, and the *p*-value for a two-tailed *t*-test with the null hypothesis *H*_0_: β_1_ = 1.

To examine the interaction between F0-modulation and reverberation we plotted template contrast in the modulated-F0 double-vowel responses against template contrast in the static-F0 double-vowel responses (Figure 12). For clarity, we plotted only those data from units having a significant template-contrast peak in both their static-F0 and modulated-F0 responses for a given level of reverberation. The effect of F0-modulation alone on the template contrast at periodicities corresponding to the two components of the double vowel, in the absence of reverberation, is quantified in Figure 12A. The solid red and blue lines indicate constrained linear least-squares fits to the data for /a/ and /i/, respectively. The slope of the linear regression in both cases is close to unity, but does differ significantly from unity (two-tailed *t*-tests: /a/, *t*_(156)_ = −9.02; /i/, *t*_(145)_ = −5.25; *p* < 0.001 for both). The addition of reverberation increases the detrimental effect of F0-modulation on template contrast at the two vowel periodicities (Figures 12B–D). This is reflected in the decreased slopes of the linear fits to the data in the reverberant conditions. In the most extreme case, with strong reverberation, only one single unit maintained a significant representation of the periodicity of /a/ in its temporal discharge pattern (Figure 12D).

**Figure 13:**
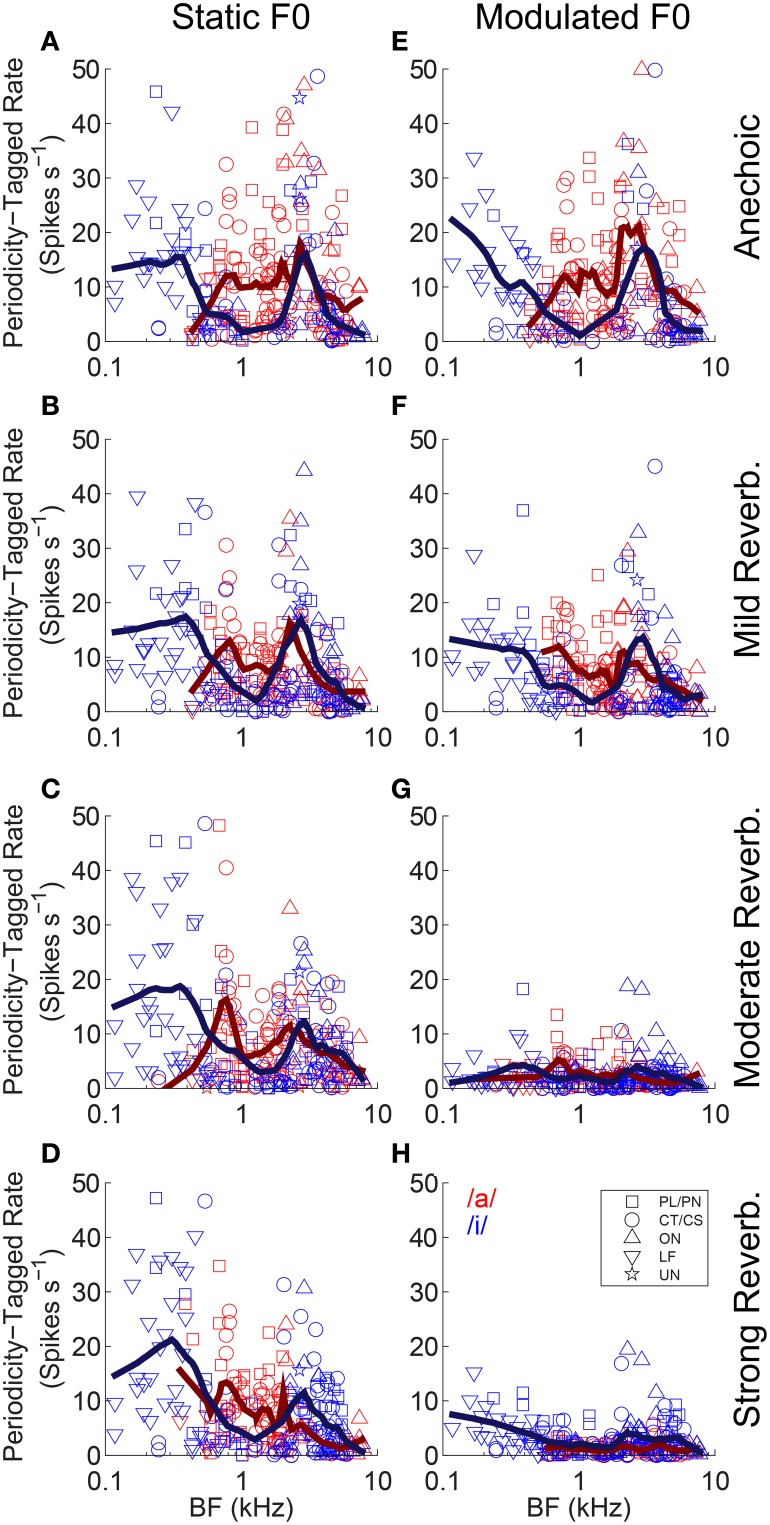
**Periodicity-tagged firing rate plotted as a function of unit BF for responses to the /a/ and /i/ components of the double vowels /a, i/ (125, 100) and /a, i/ (250, 200)**. **(A–D)** Static F0 vowels. **(E–H)** Modulated F0 vowels. *Top row* anechoic conditions. *Second row* mild reverberation. *Third row* moderate reverberation. *Bottom row* strong reverberation. **(A–H)** Red symbols indicate responses to the /a/ component. Blue symbols indicate responses to the /i/ component. Solid lines indicate *lowess* fits to the data, to demonstrate the trend across BF. Note these trend lines include responses from all unit types.

We used contrast ratios derived from the output of the pitch sieve to attempt reconstruction of the spectra of the two vowels of the double-vowel mixture. To do so, we define a *periodicity-tagged firing rate* similar to that proposed by Keilson et al. (1997). This periodicity-tagged rate for the response to the /a/ component of /a, i/ is, *R*_/*a*/_ = ([*R* · *C*_/*a*/_]/[*C*_/*a*/_ + *C*_/*i*/_]) - (0.5 · *R*), where *R* is the mean driven rate in response to the double vowel /a, i/. The periodicity-tagged rate for the response to the /i/ component of /a, i/ is then, *R*_/*i*/_ = ([*R* · *C*_/*i*/_]/[*C*_/*a*/_ + *C*_/*i*/_]) - (0.5 · *R*). Thus, defined, *R*_/*i*/_ and *R*_/*a*/_ are mutually mirror-symmetric around 0, since an increased periodicity-tagged rate to /a/, leads to a decreased periodicity-tagged rate to /i/ of equal magnitude, and *vice versa*. Therefore, we consider only the positive half of these functions, so that on this scale a periodicity-tagged rate >0 indicates the number of spikes in excess of the average number of spikes evoked which are assigned to a particular vowel of the mixture by a classifier operating on the output of the pitch-sieve stage of the analysis. If a unit's inter-spike interval distribution provided no information on which the response could be partitioned between the two vowels of the mixture, the periodicity-tagged rate for both /a/ and /i/ would be zero. Figure 13 plots the periodicity-tagged rate for both /a/ and /i/ as a function of BF and level of reverberation in the static-F0 (left column), and modulated-F0 (right column) conditions. Red symbols are periodicity-tagged spike rates in response to the /a/ component of /a, i/, and blue symbols represent the /i/ component of /a, i/. Solid lines represent local weighted regression (“*lowess*”) fits to the data (Cleveland, 1979; Cleveland and Devlin, 1988), and are shown to indicate the trend across BF. However, note these trend lines include data from all unit types.

In the static-F0 conditions, there is a strong BF-dependency on the periodicity-tagged firing rates in response to both the /a/ and /i/ components of the double vowel (Figures 13A–D). This is seen as a peak in the /i/ response at very low BFs around 0.3 kHz, with a localized minimum in the response to /i/ around 1–2 kHz, and a second peak in the periodicity-tagged rate for /i/ at BFs near 3 kHz. This corresponds approximately to the formant peaks of /i/. A similar relationship between formant frequencies and peaks in the periodicity-tagged firing rate is seen in the response to the /a/ component of the /a, i/ mixture. The clearest difference between the vowels' across-BF profiles is between their first-formant regions. Human behavioral data also suggest that ΔF0-based segregation of concurrent vowels is dominated by information in the region of the first formant (Culling and Darwin, 1993). As the level of reverberation increases, there is little change to this pattern of maxima and minima in the distribution of periodicity-tagged firing rate across the tonotopic axis in the responses to static-F0 double vowels. Compare this result with the pattern of responses to the modulated-F0 double vowels (Figures 13E–H). In the anechoic condition, there is a clear distinction between the distribution of periodicity-tagged spike rates across the BF axis for /a/ and /i/, indicating the neurons are able to segregate the two vowels of the double-vowel mixture on the basis of this coding scheme (Figure 13E). However, as reverberation is added, with increasing source-receiver distance (i.e., decreasing anechoic-to-reverberant energy ratio), the distinction between the periodicity-tagged rates for /a/ and /i/ becomes less clear (Figures 13F–H). Compare the responses to the static-F0 double vowel and the modulated-F0 double vowel in strong reverberation (Figures 13D,H). At an equal level of reverberation, the effect of reverberation on neural segregation of the two vowels of the double-vowel mixture is much greater in the modulated-F0 case than in the static-F0 case. This pattern of results is also observed in human psychoacoustic experiments. That is, ΔF0-based perceptual segregation of voiced speech sounds is impaired by the combination of reverberation and either sinusoidal F0-modulation (Culling et al., 1994), or, natural intonation (Culling et al., 2003).

## Discussion

### ΔF0-based neural segregation of concurrent vowel sounds in reverberant environments

Vowel formant peaks are represented in the across-BF mean firing-rate distribution recorded from VCN single units. For single vowels, this representation is robust to the presence of reverberation and F0 modulation, either alone or in combination. This result is consistent with previous physiological data on the rate-place representation of vowel spectra in VCN (Blackburn and Sachs, 1990; Keilson et al., 1997; Rhode, 1998; Recio and Rhode, 2000) and ANFs (Sachs and Young, 1979; Delgutte and Kiang, 1984a; Recio et al., 2002). That is, units having a BF near to a vowel's formant peak tend to pass more total energy via their peripheral filter and respond with a higher firing rate compared to units with a BF near a spectral minimum. Therefore, across BF there are peaks in the firing-rate profile corresponding to the formant energy peaks of the vowel (at least for the first two formants). Considering the temporal aspects of the spike-train responses to single-vowel sounds, we found a strong interaction between reverberation and F0 modulation on the temporal representation of single-vowel F0s. This finding is directly relatable to our previous work on the effects of reverberation on temporal representations of the F0 of harmonic complex tones with linear F0 modulation (Sayles and Winter, 2008b).

It should be noted that reverberation also reduces template contrast in response to static-F0 single vowels. The degradation in the temporal response is likely the result of randomization of the phase relationship between component partials of the vowels in the presence of reverberation. The artificial vowel sounds used here have a relatively “peaky” temporal envelope in the anechoic case, due to the phase relationship between harmonics of F0 at the output of the Klatt formant synthesizer (Klatt, 1980). With increasing reverberation strength, the direct-to-reverberant energy ratio decreases, and therefore the contribution of the reflected sound components (with random phase) is increased over the contribution from the direct sound. Inter-spike interval representations of F0-related periodicity in ANFs and VCN single units is sensitive to relative phase between component partials of complex sounds (Javel, 1980; Rhode, 1995; Cariani and Delgutte, 1996b; Sayles and Winter, 2008a). The reduced “peakiness” of the temporal envelope in the presence of reverberation leads to fewer spikes occurring *at* the F0 period, and relatively more spikes occurring *within* the F0 period. This has the effect of increasing the denominator in the calculation of template contrast at the F0 period, and therefore explains the reduced template contrast values seen in response to static-F0 single vowels in strong reverberation, compared to the same vowels under anechoic conditions.

At first glance, the reverberation-insensitive rate representation and reverberation-sensitive temporal representation of single vowels may appear to suggest that under reverberant listening conditions a rate-place representation of vowel sounds is more useful than a temporal one. However, the degraded temporal representation of single-vowel sounds has implications for the “periodicity-tagging” process for segregation of overlapping spectral envelopes of concurrent complex sounds. For double vowels, we found ΔF0-based segregation of firing rate into two separate streams fails when F0 is modulated and the vowels are heard in a reverberant environment. Our results suggest a loss of temporal information relating to the vowel F0s is an important factor underlying the detrimental effect of reverberation on perceptual segregation of competing intonated speech. The combination of F0 modulation (i.e., intonation in speech, and the basis of melody in western music) and reverberation in real-world listening spaces such as offices, living rooms, places of worship and concert auditoria likely reduces the available pitch-related information in temporal discharge patterns of peripheral auditory neurons. Without an accurate temporal representation of F0, the mechanism of “periodicity-tagging” of firing rate across frequency is impaired. This loss of F0-related temporal information in the neural responses is likely related to the physical effects of reverberation on the acoustic signal. Reverberation attenuates amplitude modulation, due to temporal smearing of the amplitude envelope, and randomization of the phase relationship between the indirect partials of a complex sound (Houtgast and Steeneken, 1973, 1985; Plomp and Steeneken, 1973). This effect is exacerbated when the harmonics of a complex sounds are frequency modulated, since the fine structure is then also smeared through time (Sayles and Winter, 2008b).

Our data were collected in response to single- and double-vowel sounds presented at one sound level (70-dB SPL). In contrast to previous studies, which have concentrated on the effects of overall sound level on the neural representation of vowel formant frequencies (e.g., Sachs and Young, 1979; Delgutte and Kiang, 1984a; Blackburn and Sachs, 1990; Recio et al., 2002), this study specifically addresses the effects of a different parameter (reverberation) on vowel representations. Due to recording-time limitations in an *in-vivo* mammalian preparation, we did not cross our reverberation parameter with an overall-level parameter. As overall level increases, the firing-rate representation of formant peaks is diminished in high-spontaneous-rate ANFs (Sachs and Young, 1979; Delgutte and Kiang, 1984a; Blackburn and Sachs, 1990). VCN chopper units are somewhat resistant to this rate-saturation effect (e.g., Blackburn and Sachs, 1990: see their Figure 16). Our choice of 70-dB SPL is consistent with a sound level at which high-spontaneous-rate ANFs show some saturation effects, but chopper units in VCN maintain a robust rate-based representation of vowels' formant structure. The restriction here to one sound level should not be taken to indicate sound level is not an important parameter to consider in the neural representation of vowels in general, and in neural segregation strategies for concurrent complex sounds (Palmer, 1990). An additional, and related, consideration is the frequency-to-place mapping on the basilar membrane, and species differences in this regard. The synthetic vowel sounds used here were modeled using parameters appropriate for the human cochlea. By scaling vowels' spectral envelopes for the frequency-place mapping of the cat cochlea, Recio et al. (2002) found the rate-place representation of vowel formants in cat ANF responses was more robust to increases in sound level. It is possible that by applying similar scaling for the guinea-pig, our rate-place profiles would be even clearer.

Reverberation-induced attenuation of temporal-envelope modulation at the output of cochlear filters likely depends on filter bandwidth and the number of harmonics interacting within the pass-band. When considering the relevance of our data to human perception it is important to take account of potential differences between the auditory periphery of humans and guinea-pigs. Some evidence supports the view that human cochlear filters are two- to three-fold sharper than in most animals (Shera et al., 2002; Oxenham and Shera, 2003; Joris et al., 2011). However, this remains a contentious issue (Ruggero and Temchin, 2005, 2007). With sharper cochlear filters, fewer harmonics interact within a filter's pass-band; i.e., the harmonics are better resolved. Sayles and Winter (2008b) found a temporal representation of the pitch of harmonic complex tones was robust to reverberation in VCN units responding to resolved harmonics. Even with the high-F0 vowels used here, it is unlikely the guinea-pig cochlea resolves more than the first harmonic (Evans, 2001; Sayles and Winter, 2010). Therefore, our results are dominated by temporal responses to envelope modulation resulting from interaction between unresolved harmonics. The periodicity of this envelope modulation is sensitive to the relative phase between the interacting harmonics.

Previous studies examined representations of noise-masked vowels in discharge patterns of ANFs (Delgutte, 1980; Sachs et al., 1983; Delgutte and Kiang, 1984b; Miller and Sachs, 1984; Geisler and Gamble, 1989) and VCN units (May et al., 1998; Winter et al., 2003). Compared to PL/PN units and ANFs, VCN CT/CS units provide a background-noise robust firing-rate representation of formant peaks (May et al., 1998). Winter et al. (2003) found a subset of onset units provide a noise-robust representation of vowel F0 in their temporal spike patterns. However, no neurophysiological study has addressed vowel segregation in the presence of background noise. The physical effects of additive noise and reverberation have some similarities, such as the random addition of (small) differences to the magnitude and phase of each component of a harmonic complex sound. Psychophysically, noise and reverberation both decrease speech intelligibility (e.g., Gelfand and Silman, 1979). However, patterns of errors in speech understanding differ substantially between noisy and reverberant environments (Nabelek, 1993; Assmann and Summerfield, 2004). Importantly, the impulse response of a room is a linear filter. As such, and in contrast to additive noise, for a steady-state source no new frequency components are added to a reverberant acoustic signal. This is a critical consideration when comparing our data to those from studies of vowel coding in background noise. We do not believe the effects of reverberation can be simply assimilated to the effects of background noise on the neural representation of complex sounds. More work is required to precisely determine the differential impact of each form of acoustic distortion on neurophysiological speech representations at peripheral stages of auditory signal processing.

### Relation to the psychoacoustics of speech segregation in rooms

Here we consider ΔF0-based *monaural* concurrent-vowel segregation, similar to previous neurophysiological studies of complex sound segregation in monaural auditory loci (Palmer, 1990; Tramo et al., 2001; Larsen et al., 2008). It is well-established that binaural hearing, resulting in release from masking for spatially-separated sources, is important for segregation of target sounds from complex maskers (Plomp, 1976; Hawley et al., 2004). However, the azimuth-difference benefit is small compared to the ΔF0-benefit for vowel segregation (Shackleton and Meddis, 1992). The azimuth-difference benefit is decreased in reverberant environments compared to anechoic conditions (Plomp, 1976; Bronkhorst and Plomp, 1990; Culling et al., 2003; Lavandier and Culling, 2007; Ruggles and Shinn-Cunningham, 2011). Decreased interaural coherence (degrading spatial segregation cues) and decreased temporal-envelope modulation both contribute to increased speech-reception thresholds in reverberant environments (Hartmann, 1983; Rakerd and Hartmann, 1985, 2005; Lavandier and Culling, 2007, 2008; Devore et al., 2009, 2010; Devore and Delgutte, 2010; Ihlefeld and Shinn-Cunningham, 2011; Monaghan et al., 2013). Consistent with theories of binaural unmasking such as equalization-cancelation (Durlach, 1972), decreased interaural coherence would increase the masking effect of interfering sounds. Our results are primarily related to the effects of reverberation on temporal-envelope modulation. Note that since the two vowels of the double-vowel mixture were convolved with identical impulse responses, this is equivalent to the two vowels originating from 0° azimuth.

The combination of reverberation and F0 modulation degrades the temporal representation of F0, and therefore limits the usefulness of this cue for neural segregation of concurrent vowels. A limitation of our data is that the two vowels of the double-vowel mixture are co-modulated; i.e., both F0s are modulated at 5 Hz, with the same amplitude and same modulation phase. We acknowledge this situation is not likely to occur frequently when listening to real-world concurrent speech. However, our stimuli are closely related to those used in human psychoacoustic studies of the interaction of F0 modulation and reverberation on double-vowel segregation, where 5-Hz (±2 semitones) co-modulated double vowels were also used (Culling et al., 1994). Furthermore, the same pattern of psychoacoustic results observed with these stimuli is also obtained with concurrent intonated speech, with each speaker having an independent F0 contour (Culling et al., 2003). Therefore, we do not expect our neurophysiological results to differ significantly with vowels having independently modulated F0s. Human behavioral studies have suggested the ΔF0 benefit for segregation is due to the auditory system exploiting the harmonic structure of the interfering vowel, to “cancel” it (Summerfield and Culling, 1992; De Cheveigné et al., 1995; Deroche and Culling, 2011). Theoretical studies have elaborated a harmonic cancelation model for segregation of competing speech sounds on the basis of hypothesized inhibitory neural delay lines (De Cheveigné, 1993, 1997, 1998). The operation of this model requires a clear representation of the periodicity of at least one vowel of a double-vowel mixture in the temporal firing pattern of peripheral auditory neurons. In the case of reverberant speech with a modulated F0, our results indicate this information can be strongly disrupted. On the basis of the residual periodicity in the VCN spike patterns we suggest either cancelation of the interferer, or enhancement of the target would be difficult. It is important to acknowledge that ΔF0 is not the only cue for perceptual segregation of complex sounds. When two vowels are presented with identical F0s (or both unvoiced), segregation performance is well above chance (Scheffers, 1983; Assmann and Summerfield, 1989). Without a ΔF0, cues such as onset asynchrony, differences in spectral-envelope shape, and spatial location can be exploited to segregate concurrent vowels (e.g., Roberts et al., 2007; Shen and Richards, 2012). Even in the presence of a ΔF0, the improvement in segregation may not be the direct result of a mechanism based on F0 *identification*; explicit pitch percepts are not necessary: harmonicity can be exploited for segregation *independent* of pitch perception *per se* (Roberts and Brunstrom, 1998, 2001).

### Effects of anesthesia

Our data were recorded from the VCN under anesthesia. Studies in an awake rabbit preparation have suggested a mechanism for compensation for the effects of reverberation on the coding of temporal-envelope modulation based on interaural-phase sensitivity in the IC (Slama, 2011; Delgutte et al., 2012). Furthermore, evidence from cortical-cooling studies suggests a role for descending connectivity in shaping the neural representation of concurrent complex sounds in the IC (Nakamoto et al., 2010). Descending connections also exist between the auditory cortex and cochlear nucleus (CN) (e.g., Schofield and Coomes, 2006). In addition, the medial olivocochlear efferent system modulates activity in CN indirectly via its action in the cochlea, and directly via synapses in CN (Liberman and Brown, 1986; Winter et al., 1989; Mulders et al., 2002; Cooper and Guinan, 2006). Therefore, it remains possible our results would be quantitatively different in the absence of anesthesia. However, although there is evidence for effects of anesthesia on responses of dorsal CN neurons, the response properties of VCN units generally do not differ between anesthetized, de-cerebrate, or awake animals (Evans and Nelson, 1973; Young and Brownell, 1976; Voigt and Young, 1980; Rhode and Kettner, 1987; May et al., 1998). In particular, the representation of vowel sounds in the awake-cat VCN does not differ substantially from the anesthetized state (May et al., 1998).

## Conclusions

Under anechoic conditions, the inter-spike interval distributions and firing rates recorded from single units in the anesthetized guinea-pig VCN support the segregation of concurrent vowel sounds. Simulating either a reverberant listening environment or intonated speech has little effect on the fundamental ability of neurons in the most peripheral of auditory brainstem processing sites to segregate complex sounds. In contrast, we find the combination of reverberation and intonation in speech sounds has profound detrimental consequences for spike-timing based neural segregation. Different VCN unit types (PL/PN, CT/CS, OC/OL/OI) behave similarly in this regard. The extent to which this effect can be mitigated by further neural processing within the ascending (and/or descending) auditory pathways remains unknown. Our data offer intriguing insight to the potential neural bases for human psychophysical phenomena in challenging acoustic environments.

## Author contributions

The study was designed by Ian M. Winter and Mark Sayles. Data were collected by Arkadiusz Stasiak, Mark Sayles, and Ian M. Winter. Data analysis was performed by Mark Sayles and Arkadiusz Stasiak, with assistance from Ian M. Winter. The manuscript was drafted by Mark Sayles, and edited for important intellectual content by Mark Sayles, Ian M. Winter and Arkadiusz Stasiak. Mark Sayles, Arkadiusz Stasiak and Ian M. Winter all approved the final edited version of the manuscript, and agree to be accountable for all aspects of the work.

### Conflict of interest statement

The authors declare that the research was conducted in the absence of any commercial or financial relationships that could be construed as a potential conflict of interest.

## Abbreviations

ANF: auditory nerve fiber
BF: best frequency
CAP: compound action potential
CN: cochlear nucleus
CS: sustained chopper
CT: transient chopper
F0: fundamental frequency
IC: inferior colliculus
LF: low-frequency
OC: onset chopper
PL: primary-like
PN: primary-like with notch
VCN: ventral cochlear nucleus.
